# Level of constitutively expressed BMAL1 affects the robustness of circadian oscillations

**DOI:** 10.1038/s41598-022-24188-4

**Published:** 2022-11-14

**Authors:** Apirada Padlom, Daisuke Ono, Rio Hamashima, Yuko Furukawa, Takashi Yoshimura, Taeko Nishiwaki-Ohkawa

**Affiliations:** 1 Laboratory of Animal Integrative Physiology, Graduate School of Bioagricultural Sciences, Nagoya University, Furo-Cho, Chikusa-Ku, Nagoya, 464-8601 Japan; 2 Department of Neuroscience II, Research Institute of Environmental Medicine, Nagoya University, Nagoya, 464-8601 Japan; 3 Department of Neural Regulation, Nagoya University Graduate School of Medicine, Nagoya, 464-8601 Japan; 4 Institute of Transformative Bio-Molecules (WPI-ITbM), Nagoya University, Furo-Cho, Chikusa-Ku, Nagoya, 464-8601 Japan

**Keywords:** Circadian rhythms, Biochemistry, Biophysics

## Abstract

The circadian rhythm is a biological oscillation of physiological activities with a period of approximately 24 h, that is driven by a cell-autonomous oscillator called the circadian clock. The current model of the mammalian circadian clock is based on a transcriptional-translational negative feedback loop in which the protein products of clock genes accumulate in a circadian manner and repress their own transcription. However, several studies have revealed that constitutively expressed clock genes can maintain circadian oscillations. To understand the underlying mechanism, we expressed *Bmal1* in *Bmal1*-disrupted cells using a doxycycline-inducible promoter and monitored *Bmal1* and *Per2* promoter activity using luciferase reporters. Although the levels of BMAL1 and other clock proteins, REV-ERBα and CLOCK, showed no obvious rhythmicity, robust circadian oscillation in *Bmal1* and *Per2* promoter activities with the correct phase relationship was observed, which proceeded in a doxycycline-concentration-dependent manner. We applied transient response analysis to the *Bmal1* promoter activity in the presence of various doxycycline concentrations. Based on the obtained transfer functions, we suggest that, at least in our experimental system, BMAL1 is not directly involved in the oscillatory process, but modulates the oscillation robustness by regulating basal clock gene promoter activity.

## Introduction

Circadian rhythms are biological oscillations of various physiological activities, such as sleep–wake cycles, hormone secretion, and metabolism, with a period of approximately 24 h. Circadian rhythms are driven by an endogenous, cell-autonomous oscillator called the circadian clock^1^. Currently, the molecular mechanism of the circadian clock is understood to be based on a transcriptional-translational negative feedback loop (TTFL), in which the translational product of clock genes represses their own transcription^1,2^. In mammals, the circadian clock is thought to consist of an essential core loop and a subsidiary ROR/REV*/Bmal1* loop, which are interlocked to generate a stable circadian oscillation^3^. In the core loop, the BMAL1-CLOCK heterodimer activates the transcription of *period* (*Per1* and *Per2)* and *cryptochrome* (*Cry1* and *Cry2*) through E-box motifs located in the promoter region^4,5^. The translational products of *Per* and *Cry* form a heterodimer that translocates into the nucleus, where it represses the transcription of their own mRNAs by inhibiting BMAL1-CLOCK function^6,7^. Thus, *Per* and *Cry* transcription and translation and the accumulation of their transcriptional and translational products oscillate in a circadian manner^8,9^. In the ROR/REV*/Bmal1* loop, *Bmal1* transcription is regulated by ROR transcriptional activators (RORα, RORβ, and RORγ)^10^ and the inhibitors REV-ERBs (REV-ERBα and REV-ERBβ)^11–13^. RORs and REV-ERBs compete for RORE regulatory elements located in the promoter region of *Bmal1*^14^, resulting in circadian oscillation in *Bmal1* transcription. Conversely, transcription of *ROR*s and *REV-ERB*s is activated by the BMAL1-CLOCK heterodimer, which also oscillates in a circadian manner^12^.

In TTFL models, oscillations in the transcriptional and translational products of clock genes are required for the circadian clock that allow cells to “tell time”^2^. However, several studies revealed that rhythmic clock gene expression is not essential for the circadian clock. For example, *Bmal1* expressed from a constitutive promoter can restore circadian oscillations in *Per2* promoter (P_*Per2*_) activity in *Bmal1*^−/−^ fibroblast cells^15^. Cell-permeant CRY1 and CRY2 proteins can rescue the arrhythmic phenotype of *Cry1*^−/−^*Cry2*^−/−^ fibroblasts when applied to culture media^16^. Furthermore, constitutive *Per2* expression in *Per1*^−/−^*Per2*^−/−^ mice restores the circadian rhythm of P_*Per2*_ activity at the cellular level, but also rescues sleep/wake cycles in the organism^17^. Therefore, it is generally believed that post-transcriptional and post-translational events play important roles in TTFL^18^. However, it is not fully understood why the constitutive clock gene expression restores circadian oscillations.

In this study, we investigated the effect of constitutive *Bmal1* expression on circadian oscillations. For simplicity, we focused on *Bmal1*, which is the only clock gene for which a single inactivation leads to loss of circadian rhythmicity^19^. We established a novel cellular system to study the effects of constitutively-expressed BMAL1 on *Bmal1* promoter (P_*Bmal1*_) activity using the firefly luciferase gene (*Fluc*) as a reporter. Endogenous *Bmal1* was inactivated in human U2OS cells containing the P_*Bmal1*_::*Fluc* reporter and an exogenous gene encoding Myc-tagged BMAL1 (MYC-BMAL1) driven by a non-rhythmic, doxycycline (DOX)-inducible promoter was stably introduced. Using these cells, we investigated the effect of constitutively-expressed MYC-BMAL1 on P_*Bmal1*_ and P_*Per2*_ activity and on the accumulation of proteins involved in the ROR/REV*/Bmal1* loop. Finally, we performed transient response analysis to obtain transfer function models that recapitulate the behavior of P_*Bmal1*_ under variable MYC-BMAL1 induction.

## Results

### *Bmal1* promoter activity at various doxycycline concentrations

**Figure 1:**
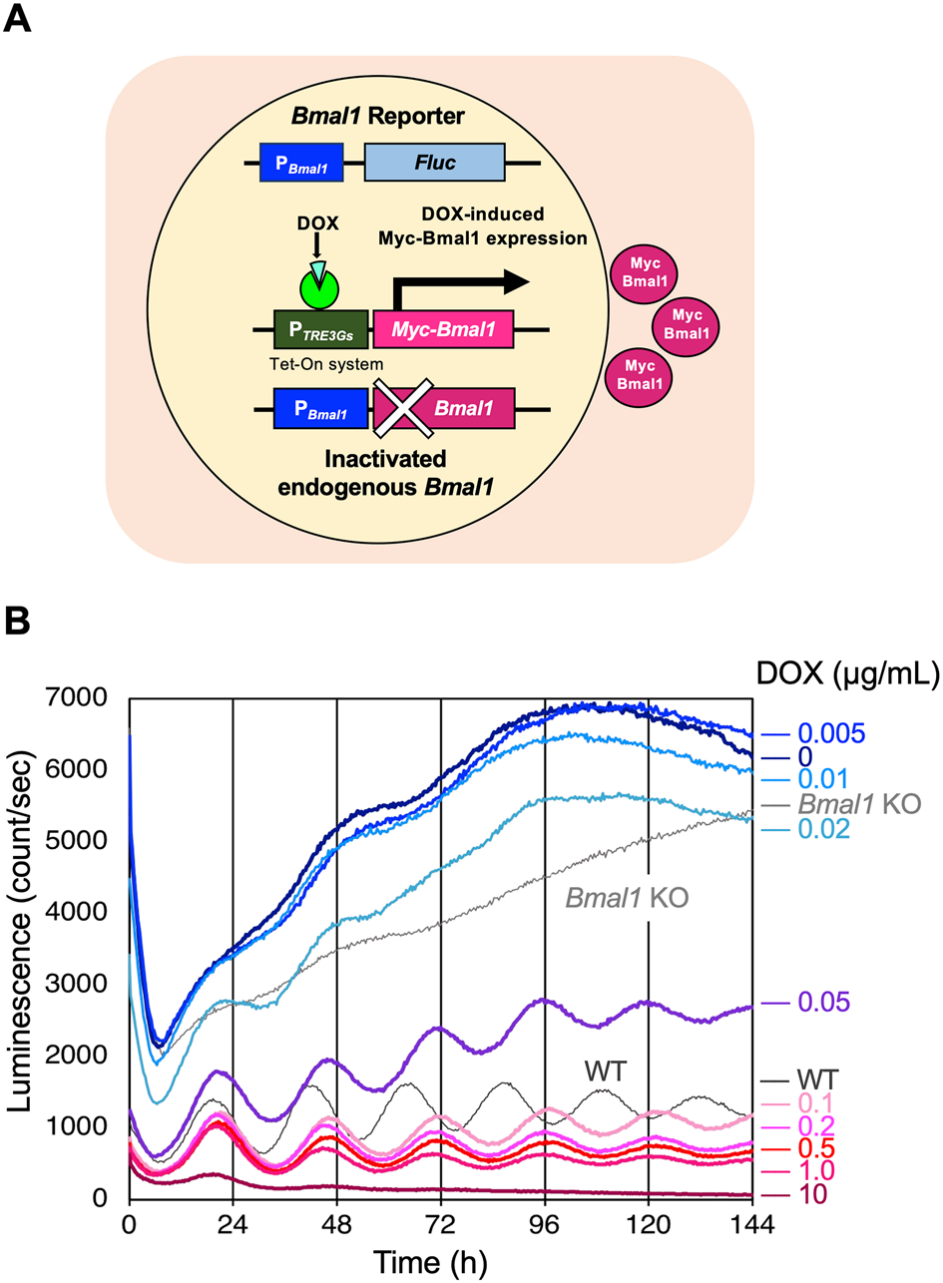
MYC-BMAL1 expression driven by a DOX-inducible promoter restores rhythmic *Bmal1* promoter activity. (**A**) Schematic diagram of U2OS-P_*Bmal1*_::*Fluc*/Δ*Bmal1*/P_*TRE3Gs*_::*Myc-Bmal1* strains. Endogenous *Bmal1* was inactivated by CRISPR-Cas9 and a gene coding MYC-BMAL1 was expressed from the DOX-inducible promoter P_*TRE3Gs*_. *Bmal1* promoter activity was monitored using the P_*Bmal1*_::*Fluc* reporter. We obtained seven strains (-2, -17, -23, -27, -33, -51, and -59). (**B**) Time course of luminescence measurements from the P_*Bmal1*_::*Fluc* reporter in strain-2. Measurements were performed every 20 min and were taken in triplicate. The average values are shown. DOX concentrations are indicated on the right side of the graph.

We developed novel P_*Bmal1*_::*Fluc* reporter cell lines in which endogenous *Bmal1* was inactivated by CRISPR-Cas9 and MYC-BMAL1 expression was driven by P_*TRE3Gs*_, a DOX-inducible promoter (Fig. 1A). We obtained seven cell lines and measured their luminescence from P_*Bmal1*_::*Fluc*. At time 0, 100 nM dexamethasone was added to reset the circadian clock^20^ and luminescence was measured. The overall intensity of luminescence decreased as the DOX concentration increased, suggesting that BMAL1 protein repressed P_*Bmal1*_ activity (Fig. S1) either directly or indirectly. Consistently, Yu et al. previously reported that BMAL1 protein represses *Bmal1* transcription^21^. The P_*Bmal1*_ activity oscillation amplitudes differed among the seven cell lines (Fig. S1). In strains -2 and -51, robust circadian oscillations were restored by 0.1 and 1 µg/mL DOX. Rhythmicity was restored in strains -33 and -59, but not as robustly as in strains -2 and -51. In strains-17, -23, and -27, the P_*Bmal1*_ rhythmicity was unclear. The robust rhythmicity can be explained, at least in part, by the MYC-BMAL1 accumulation (Fig. S2, see Discussion).

Strain-2 was selected for further experiments. Luminescence from P_*Bmal1*_::*Fluc* was strongly affected by the DOX concentration (Fig. 1B). From 0–0.02 µg/mL DOX, luminescence from P_*Bmal1*_ fluctuated and oscillation was weak. In the presence of 0.05–0.1 µg/mL DOX, the luminescence gradually increased with clear damped oscillations. From 0.2–1.0 µg/mL DOX, the luminescence showed damped oscillation gradually stabilized at a value specific to each DOX concentration. At 10 µg/mL DOX, the luminescence stabilized after a single overshoot. These results suggest that the amount of MYC-BMAL1 affects the baseline and robustness of P_*Bmal1*_ activity oscillations. We also observed that the DOX concentration had little effect on the period, which was calculated to be approximately 25 h using chi-square periodogram analysis at any DOX concentration (Table 1 and Fig. S3).

**Table 1:** Period of P_*Bmal1*_::*Fluc* oscillation calculated for each DOX concentration.

DOX concentration (µg/mL)	10	1	0.1	0.05	0.02	0.01
Period (τ) (h)	25.7	25.0	25.3	25.0	24.7	N.D

Data from 0 to 144 h shown in Fig. 1B were detrended using 6th-order polynomials and subjected to a chi-square periodogram analysis using Lumicycle analysis software (ActiMetrics; Wilmette, IL, USA).

### Simultaneous measurements of *Bmal1* and *Per2* promoter activity

**Figure 2:**
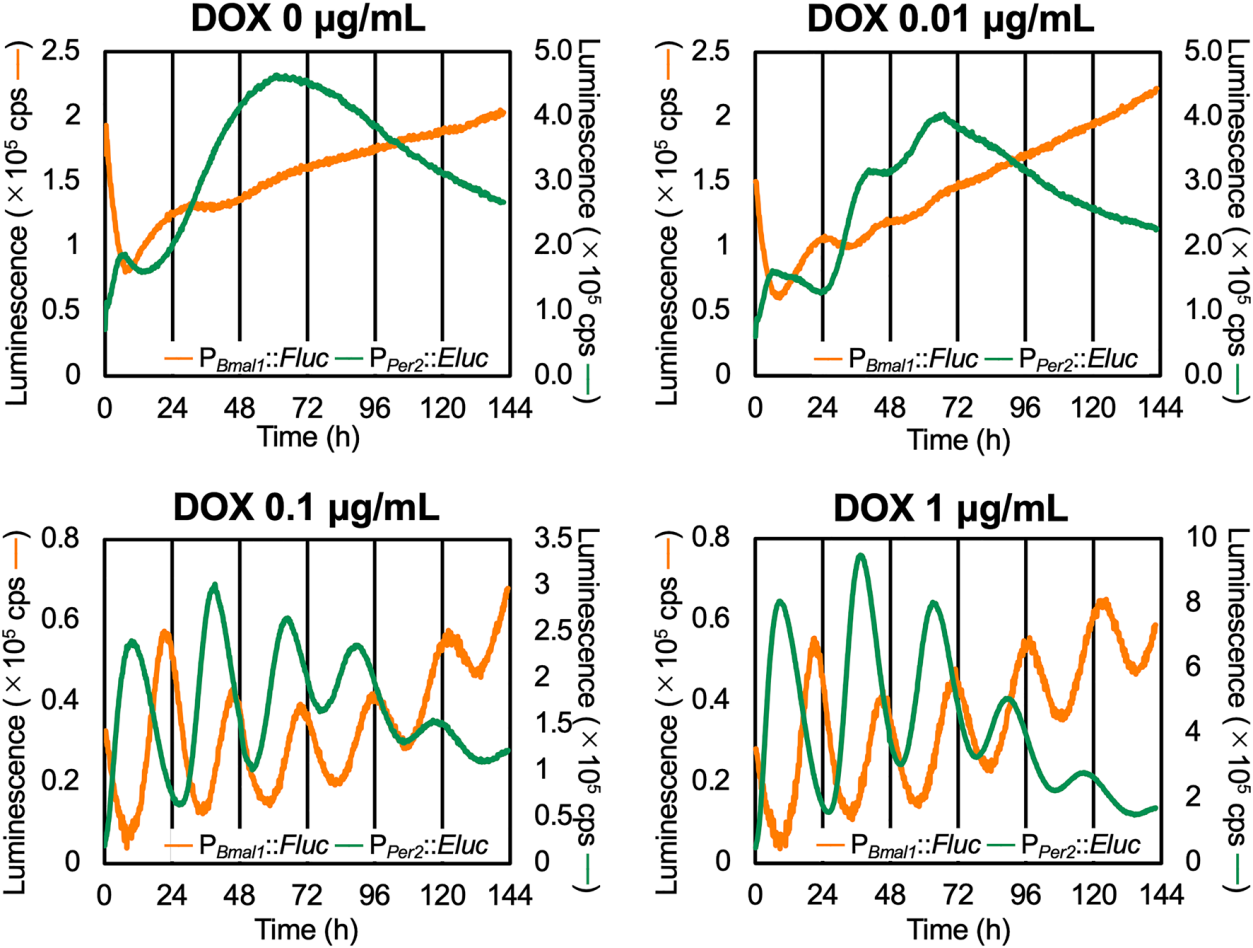
Constitutively expressed MYC-BMAL1 restores circadian rhythmicity in both P_*Bmal1*_ and P_*Per2*_ activity. U2OS-P_*Bmal1*_::*Fluc*/Δ*Bmal1*/P_*TRE3Gs*_::*Myc-Bmal1* strain-2 cells were transiently transfected with the P_*Per2*_::*Eluc* reporter and were treated with different concentrations of doxycycline (DOX). Dual wavelength luminescence measurements of P_*Bmal1*_::*Fluc* and P_*Per2*_::*Eluc* were performed for 6 days. Data presented are the representative curves of 3 independent measurements. The orange lines show luminescence from P_*Bmal1*_::*Fluc* and the green lines show luminescence of P_*Per2*_::*Eluc*.

To examine whether constitutive MYC-BMAL1 expression restores circadian oscillation in P_*Per2*_ activity, we transiently introduced an Emerald Luc reporter driven by P_*Per2*_ (P_*Per2*_::*Eluc*) into strain-2. Dual-wavelength measurements of P_*Bmal1*_::*Fluc* and P_*Per2*_::*Eluc* were performed as previously described^22^ in the presence of various DOX concentrations (Fig. 2). Overall, DOX affected P_*Bmal1*_ and P_*Per2*_ activity in a similar manner. At 0.1 and 1 µg/mL DOX, clear damped P_*Bmal1*_ and P_*Per2*_ oscillations were detected, while at 0 and 0.01 µg/mL DOX, the baseline luminescence from P_*Bmal1*_ and P_*Per2*_ reporters was highly unstable. We could not obtain luminescence data at 10 µg/mL DOX, possibly because simultaneous treatment with the transfection reagent and a high DOX concentration decreased cell viability. The oscillation periods of P_*Per2*_ and P_*Bmal1*_ activities were not significantly affected by the DOX concentration (Table 2 and Fig. S3). Notably, the antiphase relationship between P_*Bmal1*_ and P_*Per2*_ previously observed in wild-type cells^12^ was recapitulated.

**Table 2:** Period of P_*Bmal1*_::*Fluc* and P_*Per2*_::*Eluc* oscillation calculated for each DOX concentration.

DOX concentration (µg/mL)	1	0.1	0.01	0
Period (τ) of P_*Bmal1*_::*Fluc* (h)	24.8	24.5	N.D	N.D
Period (τ) of P_*Per2*_::*Eluc* (h)	26.8	26.7	26.5	N.D

The 0 to 141 h data shown in Fig. 2 were detrended by 6th-order polynomials and subjected to chi-square periodogram analysis using Lumicycle analysis software (ActiMetrics; Wilmette, IL, USA).

### Measurement of the levels of translational and transcriptional products of exogenous *Bmal1*

**Figure 3:**
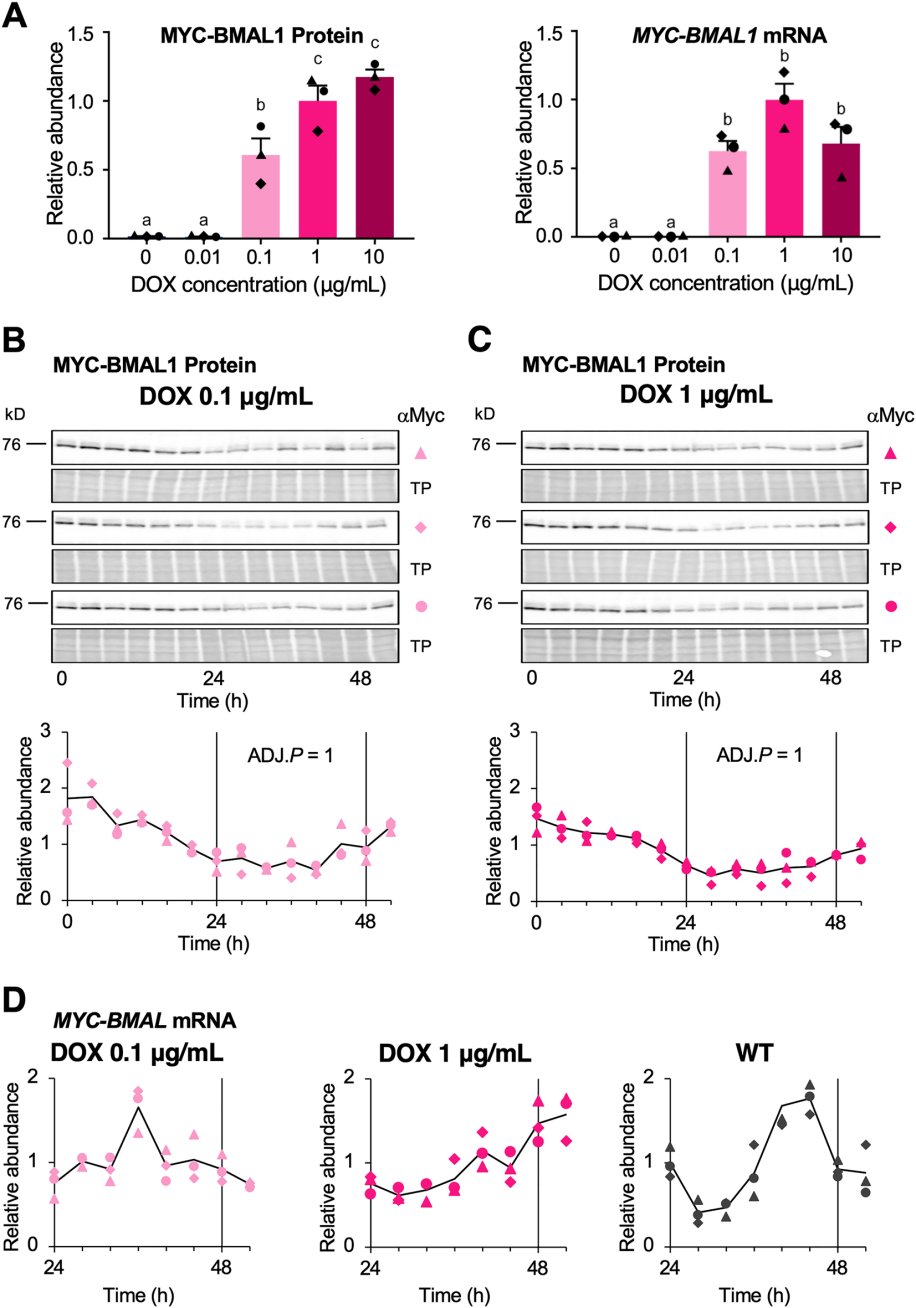
MYC-BMAL1 protein and mRNA accumulation do not show significant circadian rhythmicity. Total protein and RNA samples were collected from U2OS-P_*Bmal1*_::*Fluc*/Δ*Bmal1*/P_*TRE3Gs*_::*Myc-Bmal1* strain-2 cell cultures every 4 h from 0 to 52 h (for total protein) or 24 to 52 h (for total RNA) after adding 100 nM dexamethasone. (**A**) Graphs showing relative MYC-BMAL1 protein (left panel) and mRNA accumulation (right panel) after treatment with different concentrations of doxycycline (DOX). For protein accumulation, equal amounts of the samples collected for each DOX concentration were mixed and subjected to immunoblot analysis using anti-Myc antibody. For mRNA accumulation, the average of all the time points for each DOX concentration was calculated. Results were normalized using the average values at 1 µg/mL DOX. Data are shown as the mean ± SEM. N = 3 samples/group, one-way ANOVA followed by Tukey’s multiple comparison test. Different characters (a, b, c) indicate significant differences (*P* < 0.05). (**B**,**C**) Time course of MYC-BMAL1 protein expression after addition of dexamethasone (time 0) in the presence of 0.1 µg/mL (**B**) and 1 µg/mL DOX (**C**). Protein samples were collected and subjected to immunoblot analysis in three independent experiments (upper panels). Markers (filled traingle, filled diamond, and filled circle) indicate MYC-BMAL1 bands in three biological replicates, and TP indicates total protein stains. Graphs (lower panels) show the quantification of MYC-BMAL1 amount by densitometry. The intensity of each band was normalized by total protein, and values were normalized using the average of all time points in each series. Black lines indicate the average values of the three biological replicates. No significant rhythmicity in the circadian range (between 20 to 28 h) was detected in the presence of 0.1 and 1 µg/mL DOX (JTK cycle test, ADJ.*P* = 1). (**D**) Time course of *MYC-BMAL1* mRNA expression after addition of dexamethasone (time 0). Total RNA was extracted from strain-2 cells in the presence of 0.1 and 1 µg/mL DOX and from wild-type U2OS cells. Samples were analyzed by quantitative reverse-transcription PCR. Relative expression was calculated using Pfaffl’s method^36^ with *GAPDH* as an internal control. Markers (filled triangle, filled diamond, and filled circle) indicate three biological replicates. Values were normalized using the average of all time points in each series. Black lines indicate the average values of the three biological replicates.

Next, we measured MYC-BMAL1 protein and mRNA levels in the presence of DOX. We collected samples of total protein and total RNA from strain-2 every 4 h after stimulation with 100 nM dexamethasone and subjected them to immunoblot analysis and quantitative RT-PCR. To examine DOX-induced changes in the overall MYC-BMAL1 levels, we analyzed an equal-volume mixture of samples from 0 to 52 h after stimulation. The total amount of MYC-BMAL1 increased remarkably between 0.01 and 0.1 µg/mL of DOX (Fig. 3A, left panel), the same concentration range at which baseline stability and robustness of the oscillation markedly increased (Fig. 1B). The average MYC-BMAL1 mRNA levels from 24 to 52 h showed the same tendency (Fig. 3A, right panel). We then assessed MYC-BMAL1 accumulation in the presence of 0.1 and 1 µg/mL DOX over time (Fig. 3B,C). Unlike the luminescence traces from P_*Bmal1*_::*Fluc*, no significant rhythmicity in the circadian range (between 20 and 28 h) was observed for MYC-BMAL1 accumulation (JTK cycle test, adjusted (ADJ.) *P* = 1)^23^. We also measured the changes in mRNA levels from 24–52 h after DOX addition using quantitative RT-PCR (Fig. 3D). The endogenous *Bmal1* transcript from wild-type U2OS cells showed clear daily fluctuations, with its peak and trough coinciding with those of P_*Bmal1*_ activity (Fig. 1B, WT). However, no such relationship was observed for *MYC-BMAL1* mRNA in the presence of 0.1 and 1 µg/mL DOX.

We also compared the average levels of DOX-induced MYC-BMAL1 protein in pooled strain-2 samples collected from 0–52 h at 4-h intervals with that of endogenous BMAL1 expressed in wild-type U2OS cells (Fig. S4). The amount of endogenous BMAL1 was about 25% of that of MYC-BMAL1 induced by 1 µg/mL DOX, indicating that higher *BMAL1* expression is required when it is driven by a non-rhythmic promoter, suggesting the biological importance of rhythmic promoters.

### Accumulation of proteins involved in the ROR/REV*/Bmal1* loop

**Figure 4:**
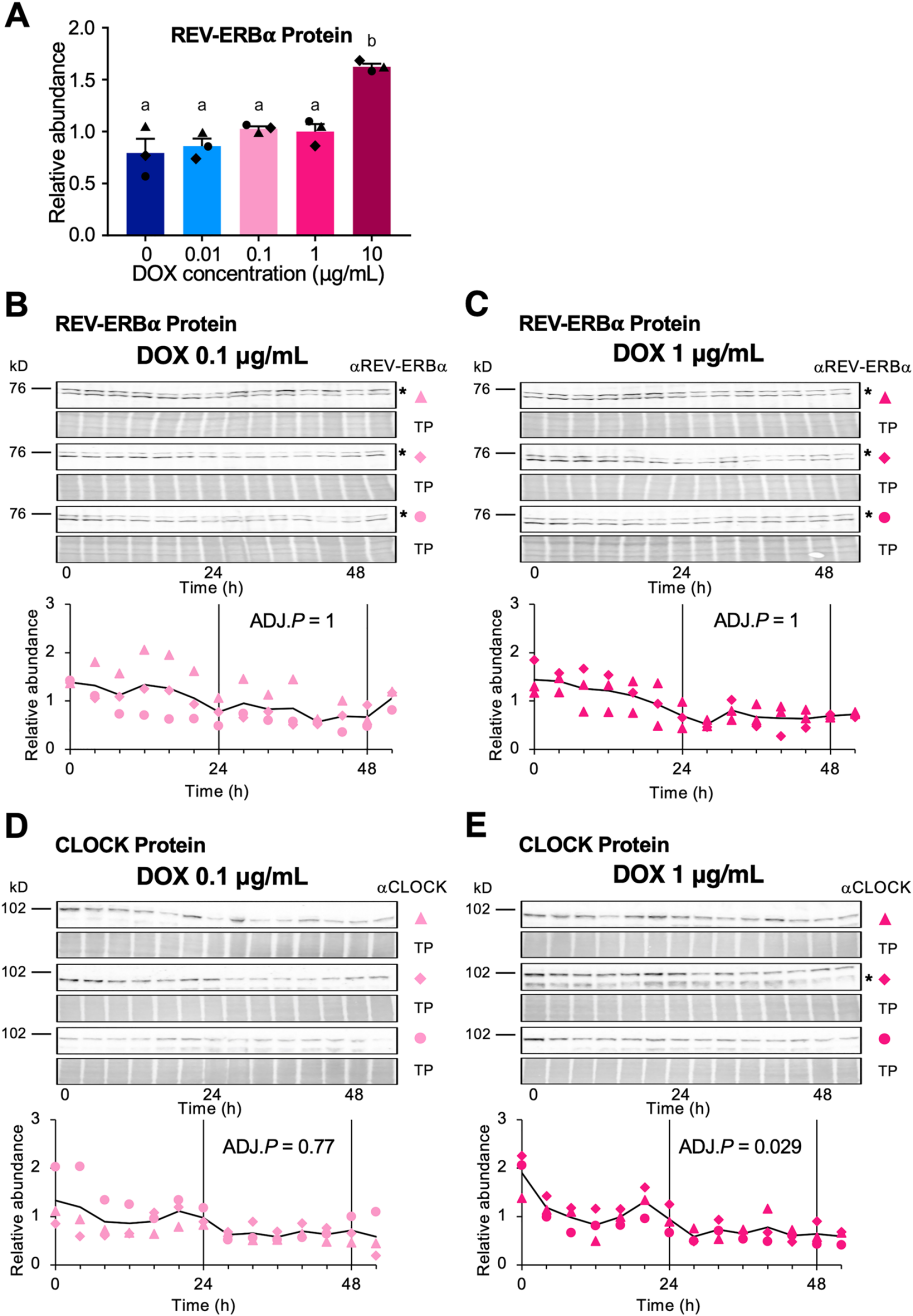
Accumulation of REV-ERBα and CLOCK does not show obvious circadian rhythmicity. (**A**) A mixture of equal amounts of protein samples collected from U2OS-P_*Bmal1*_::*Fluc*/ΔBmal1/P_*TRE3Gs*_::Myc-Bmal1 strain-2 cell cultures from 0 to 52 h after the addition of 100 nM dexamethasone in the presence of each doxycycline (DOX) concentration was subjected to immunoblot analysis using anti-REV-ERBα antibodies. The amount of protein was calculated by the density of each band versus total protein and were normalized using the value from the 1 µg/mL DOX group. The results are shown as mean ± SEM (N = 3). Different characters (a, b) indicate significant differences (one-way ANOVA followed by Tukey’s multiple comparison test, *P* < 0.05). (**B**–**E**) Time course of protein expression in the presence of 0.1 µg/mL (**B**,**D**) and 1 µg/mL DOX (**C**,**E**). Images showing the protein bands detected for REV-ERBα (**B**–**C**), CLOCK (**D**–**E**), and total protein (TP) stains (upper panels). Asterisks in (**B**,**C**,**E**) indicate nonspecific bands. Markers (filled traingle, filled diamond, and filled circle) indicate three biological replicates. For REV-ERBα detection, the same blots used for MYC-BMAL1 detection were reprobed. The protein amount was quantified using densitometry (lower panel). The relative expression of REV-ERBα (**B**–**C**) and CLOCK (**D**–**E**) protein was calculated by the density of each band vs. total protein and were normalized against the intensity of pooled 0 to 52 h samples. Black lines indicate the average values of three biological replicates. No significant rhythmicity was detected for REV-ERBα at 0.1 and 1 µg/mL DOX (JTK cycle test, ADJ.*P* = 1) or CLOCK at 0.1 µg/mL DOX (JTK cycle test, ADJ.*P* = 0.77). CLOCK at 1.0 µg/mL DOX exhibited significant rhythmicity (JTK cycle test, ADJ.*P* = 0.029) with low amplitude (JTK cycle test, AMP = 0.23).

In the current model of the mammalian circadian clock, BMAL1, CLOCK, REV-ERBs, and RORs constitute the ROR/REV*/Bmal1* loop^10–14^. To examine how constitutive MYC-BMAL1 expression affects CLOCK and REV-ERBα accumulation, we performed immunoblot analysis using protein samples collected in the presence of different DOX concentrations over time. The overall protein levels of REV-ERBα measured in pooled samples collected 0–52 h after stimulation showed an increasing trend at higher DOX concentrations (Fig. 4A). However, in contrast to MYC-BMAL1 expression (Fig. 3A), no significant change was observed between 0.01 and 0.1 µg/mL DOX. We also examined changes in the REV-ERBα (Fig. 4B,C) and CLOCK (Fig. 4D,E) in the presence of 0.1 and 1 µg/mL DOX over time. No significant rhythmicity in the circadian range was observed, except for CLOCK at 1 µg/mL DOX using the JTK cycle test (ADJ.*P* = 0.029 for CLOCK at 1 µg/mL, 0.77 for CLOCK at 0.1 µg/mL, and ADJ.*P* = 1 for REV-ERBα). The CLOCK oscillation amplitude was 0.23 at 1 µg/mL DOX using the JTK cycle test, suggesting that the rhythmicity of CLOCK accumulation, if any, was very weak.

### Transient response analysis of P_*Bmal1*_ activity

To elucidate the mechanism underlying the generation of P_*Bmal1*_ circadian oscillations under constitutive MYC-BMAL1 expression, we performed transient response analysis. This method identifies a transfer function (*i.e.*, an input–output relationship of a linear system) from the observed data to reveal the underlying system dynamics^24,25^. In our framework, dexamethasone administration was considered the input to stimulate P_*Bmal1*_ activity. The transfer functions of the stimulated transient P_*Bmal1*_ activity were estimated using the system identification toolbox pre-installed in MATLAB (version R2019b; MathWorks, Natick, MA, USA). The administration of 100 nM dexamethasone at time 0 was approximated by a unit step input to the system, which can be expressed by the following unit step function:

1 $$u\left( t \right) = \left\{ {\begin{array}{*{20}c} {0 (t < 0)} \\ {1 \left( {t \ge 0} \right)} \\ \end{array} } \right..$$

Denoting $U\left( s \right)$ and $Y\left( s \right)$ as the Laplace transforms of the input and output signals, respectively, the transfer function G(s) is represented as:

2 $$G\left( s \right) = \frac{Y\left( s \right)}{{U\left( s \right)}}.$$

**Figure 5:**
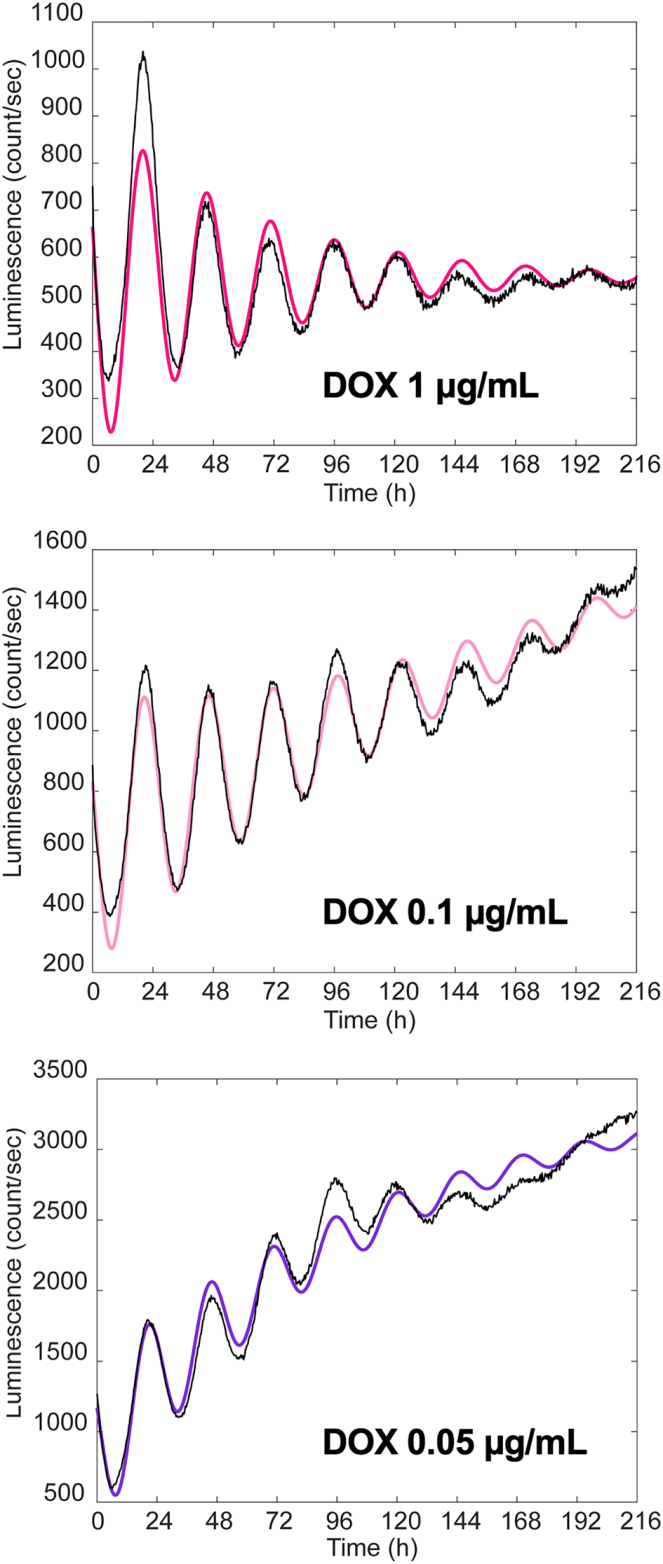
Transfer functions reproducing the behavior of P_*Bmal1*_ activity obtained by transient response analysis. Luminescence data of P_*Bmal1*_ activity in the presence of 0.05, 0.1 and 1 µg/mL doxycycline (DOX) shown in Fig. 1B were subjected to transient response analysis. Experimental values of luminescence intensity are shown in black and simulated data are shown in red for 1 µg/mL DOX (upper panel), pink for 0.1 µg/mL DOX (middle panel), and purple for 0.05 µg/mL DOX (bottom panel).

As shown in Fig. 5, P_*Bmal1*_ activity in the presence of 1 µg/mL DOX was approximated by the following transfer function with two poles and no zeros:

3 $$G\left( s \right) = \frac{{b_{0} }}{{s^{2} + a_{1} s + a_{0} }}.$$

The coefficients were estimated as $a_{1} = 0.03214$, $a_{0} = 0.06171$, and $b_{0} = 34.42$. This formula can be rewritten as the following second-order system representing a damped oscillator:

4 $$G\left( s \right) = K \cdot \frac{{\omega_{n}^{2} }}{{s^{2} + 2\zeta \omega_{n} s + \omega_{n}^{2} }}.$$

where ω_n_, ζ, and *K* are the natural angular frequency, damping coefficient, and gain, respectively. Using ω_n_ and ζ, the period τ of the damped oscillator is determined as:

5 $$\tau = \frac{2\pi }{{w_{n} \sqrt {1 - \zeta^{2} } }}.$$

On the other hand, P_*Bmal1*_ activity in the presence of 0.05 and 0.1 µg/mL DOX were approximated by the following third-order transfer function with three poles and no zeros (Fig. 5):

6 $$G\left( s \right) = \frac{{b_{0} }}{{\left( {s^{2} + a_{1} s + a_{0} } \right)\left( {c_{0} s + 1} \right)}} .$$

The coefficients were estimated as $a_{1} = 0.02363$, $a_{0} = 0.06411$, $b_{0} = 224.6$, $c_{0} = 118.4$ for 0.05 µg/mL DOX; and $a_{1} = 0.01804$, $a_{0} = 0.06051$, $b_{0} = 75.58$, $c_{0} = 350.2$ for 0.1 µg/mL DOX. This third-order transfer function can be decomposed into first-order and second-order systems, as follows:

7 $$G\left( s \right) = K \cdot \frac{1}{{T_{s} s + 1}} \cdot \frac{{\omega_{n}^{2} }}{{s^{2} + 2\zeta \omega_{n} s + \omega_{n}^{2} }}.$$

In a first-order system (*i.e.*, $\frac{1}{{T_{s} s + 1}}$), the time constant $T_{s}$ characterizes the response time required for the baseline luminescence signal to rise exponentially to its steady state. The parameter values calculated for each DOX concentration are listed in Table 3.

**Table 3:** Parameters of oscillation calculated for each DOX concentration.

DOX concentration (µg/mL)	1	0.1	0.05
Natural angular velocity (ω_n_) (h^−1^)	0.2484	0.2460	0.2532
Damping coefficient (ζ) (h^−1^)	0.06469	0.03668	0.04667
*Period (τ) (h)	25.4	25.6	24.8
Time constant (T_s_) (h^−1^)	–	350.2	118.4
Gain (K)	557.8	1249	3504

*Calculated by substituting the ω_n_ and ζ values into Eq. (5).

This analysis indicated that in the presence of 0.01 µg/mL DOX, a higher-order transfer function was required to describe the behavior of P_*Bmal1*_ activity, which is difficult to interpret using a combination of basic elements (Fig. S5). It is possible that the baseline P_*Bmal1*_ activity became uncontrollable when the Myc-BMAL1 concentration was too low.

## Discussion

TTFL is the model of choice for explaining circadian oscillations in clock gene transcription^1,2^. However, this model cannot fully explain why constitutive clock gene expression restores circadian oscillations. In this study, we performed quantitative experiments using a newly established P_*Bmal1*_::*Fluc* reporter cell line in which endogenous *Bmal1* is inactivated by CRISPR-Cas9 and exogenous *Bmal1* is expressed under a DOX-inducible promoter. This in vitro system allowed us to reproduce the restored circadian oscillations under constitutive *Bmal1* expression, as previously reported^15^.

We established seven cell lines, strains-2, -17, -23, -27, -33, -51, and -59 and measured luminescence from the P_*Bmal1*_::*Fluc* reporter (Fig. S1) induced by DOX treatment two days before synchronization with dexamethasone. Among these, strains-2, and -51 restored the robust circadian oscillations in the presence of 0.1 and 1 µg/mL DOX. Strains-59 and -33 also restored rhythmicity, but to a lesser extent. Strains-17, 23, and 27 exhibited no obvious oscillations. These differences can be explained, at least partially, by the amount of induced MYC-BMAL1. We compared MYC-BMAL1 levels in the seven strains treated with 1 or 0.01 µg/mL DOX for 2 days (Fig. S2). In the presence of 1 µg/mL DOX (Fig. S2A), MYC-BMAL1 levels were significantly higher in strains-23 and -33 and lower in strain-27 and 33 than in strain-2. Strains -51 and -59 showed no significant differences compared to strain-2. In strain-23, MYC-BMAL1 was three-to four-fold higher than in strain-2, while in strain-33, the level was comparable to that in strain-51. These results indicate that an appropriate induction level of MYC-BMAL1 is required to restore rhythmicity. In strain-51, weak rhythmicity was observed, even in the presence of 0.01 µg/mL DOX, which may reflect the slightly higher MYC-BMAL1 accumulation compared to strain-2 at this concentration (Fig. S2B).

Our results indicate that exogenous BMAL1 expressed by a non-rhythmic DOX-inducible promoter can restore circadian rhythmicity in *Bmal1*-disrupted cells. P_*Bmal1*_ and P_*Per2*_ activity exhibited robust circadian rhythms in the presence of 0.1 and 1 µg/mL DOX, whereas the accumulation of MYC-BMAL1 and REV-ERBα at 0.1 and 1 µg/mL DOX, and CLOCK at 0.1 µg/mL DOX did not show significant circadian rhythmicity. The rhythmicity of CLOCK accumulation at 1 µg/mL DOX was significant but quite weak, with an amplitude of 0.23, as estimated by JTK cycle tests. We expect that this CLOCK rhythmicity is not the cause of the P_*Bmal1*_ oscillation for the following reasons: (i) to our knowledge, no reports have been published demonstrating that CLOCK regulates *Bmal1* transcription by directly binding to the regulatory region of *Bmal1*; (ii) the translational products of *REV-ERB*s and *ROR*s, whose expression is regulated by the CLOCK-BMAL1 heterodimer, regulate *Bmal1* transcription by directly binding to the regulatory region of *Bmal1*. However, as shown in Fig. 4, the REV-ERBα did not show significant oscillations at 0.1 and 1 µg/mL DOX while it exhibited clear circadian oscillation in WT U2OS (Fig. S6). Therefore, robust circadian oscillations at the promoter level are unlikely to be driven by oscillations at the protein level, although this possibility cannot be completely excluded.

CLOCK, BMAL1, and REV-ERBα undergo circadian changes in phosphorylation state^26,27^. In particular, CLOCK and BMAL1 phosphorylation plays essential roles in circadian oscillations by regulating protein–protein interactions, nuclear localization, and transcriptional activity^26,28,29^. Phosphorylated proteins are often detected by electrophoretic mobility shifts during SDS-PAGE^30^. However, we did not detect a remarkable mobility shift of the CLOCK, BMAL1, and REV-ERBα bands over time (Figs. 3 and 4). Therefore, it is improbable that rhythmic P_*Bmal1*_ and P_*Per2*_ activity is driven by circadian phosphorylation of these proteins. However, we cannot completely rule out this possibility because a small fraction of these proteins, such as the nuclear BMAL1-CLOCK heterodimer, may exhibit circadian oscillations^28^.

Because the components of the core loop were not genetically manipulated in our *Bmal1*-inducible cell line, it is possible that the oscillation in P_*Bmal1*_ activity is driven by the intact core loop. However, this does not seem to be the case because the amplitude of the P_*Bmal1*_ and P_*Per2*_ oscillations was simultaneously restored in accordance with the DOX concentration (Fig. 2). These results suggest that induced BMAL1 affects P_*Bmal1*_ and P_*Per2*_ activity equally and that no hierarchical relationship exists between P_*Bmal1*_ and P_*Per2*_. Furthermore, P_*Bmal1*_ oscillations were almost antiphase with P_*Per2*_ activity, as in wild-type cells, even though functional *Bmal1* was not driven by an endogenous promoter containing RORE. It is difficult to explain the behavior of P_*Bmal1*_ and P_*Per2*_ observed in our experiment using the current TTFL model.

The oscillation periods of P_*Bmal1*_ and P_*Per2*_ were calculated to be approximately 25 and 27 h, respectively. A similar difference was reported in ex vivo suprachiasmatic nucleus (SCN) culture^31^, suggesting that the core loop and ROR/REV*/Bmal1* loop oscillate independently. In our experimental system, P_*Bmal1*_ and P_*Per2*_ activity might be governed by independent oscillators.

In the past two decades, numerous mathematical models describing circadian systems have been proposed^32^. In these models, TTFL behaviors, such as the activation and repression of clock gene transcription by clock gene products, are expressed using a set of differential equations containing experimentally determined parameters. Mirsky et al. mimicked constitutive *Bmal1* expression by setting *Bmal1* mRNA synthesis to a fixed rate^33^. This model predicted that the levels of transcriptional and translational products of *Per*, *Cry, Clock, ROR*c, and *Rev-erbα*, and the translational product of *Bmal1*, exhibited clear circadian oscillations even under constant *Bmal1* mRNA levels. In contrast, we did not observe clear rhythmicity in BMAL1, CLOCK, or REV-ERBα protein levels under constitutive *Bmal1* expression. Relogio et al. demonstrated that the amplitude of REV-ERB and ROR oscillations and their phase relationship are crucial for generating *Bmal1* transcriptional oscillations in the correct phase relative to other clock genes^34^. However, our results showed that although REV-ERBα levels were almost constant, P_*Bmal1*_ activity oscillated in the correct phase relative to P_*Per2*_. Therefore, it is difficult to interpret our results using these mathematical models.

To gain insight into the mechanisms driving the circadian oscillation of P_*Bmal1*_ and P_*Per2*_ promoter activity, we performed transient response analysis^24,25^. At 1 µg/mL DOX, the experimental data were well approximated by a second-order system that represented a damped oscillation. When the DOX concentration was lowered to 0.1 and 0.05 µg/mL, the luminescence from the P_*Bma1*_::*Fluc* was approximated by a third-order system that can be interpreted as a damped oscillation forced through a first-order system. For all three cases, the damping coefficient (ζ) was quite small (*i.e.*, less than 0.07), implying that the damping effect was rather weak. The oscillation periods (τ) were all in a similar range between 24.8 and 25.6 h (Table 3). These results suggest that the amount of BMAL1 does not markedly affect the oscillatory parameters, but has a major impact on the baseline P_*Bmal1*_ activity. Our analysis presents the possibility that a weakly damped oscillator system, whose molecular mechanism is yet to be clarified but is almost independent of *BMAL1*, underlies the circadian clock mechanism. Our results also suggest that this oscillator regulates P_*Bma1*_ and P_*Per2*_ activities in parallel.

Based on our results, it is possible that in our experimental system, the roles of *Bmal1* in circadian oscillations are different from those assumed in the TTFL models. Further studies are required to determine whether the function of *Bmal1* described in this study is specific to our experimental system or whether it is true for the mammalian circadian system.

## Materials and methods

### Disruption of ***BMAL1*** in U2OS-P_***Bmal1***_::***Fluc*** cells

Human U2OS cells (HTB-96; American Type Culture Collection, Manassas, VA, USA) containing the P_*Bmal1*_::*Fluc* reporter (U2OS-P_*Bmal1*_::*Fluc*)^35^ were plated in 35 mm culture dishes (Nunc EasYDish; Thermo Fisher Scientific, Waltham, MA, USA) at a density of 2 × 10^5^ cells/dish in Dulbecco’s modified Eagle’s medium (DMEM) (D6429; Sigma-Aldrich, St. Louis, MO, USA) supplemented with 10% fetal bovine serum, 2 mM glutamine, 100 U/mL penicillin, and 100 µg/mL streptomycin. The cells were cultured at 37 °C with 10% CO_2_ for approximately 24 h.

U2OS-P_*Bmal1*_::*Fluc* was transfected with 1.4 µg of human *BMAL1* CRISPR/Cas9 KO plasmid (sc-400808; Santa Cruz Biotechnology, Dallas, TX, USA) consisting of a pool of three plasmids, each encoding the Cas9 nuclease, a target-specific 20 nt guide RNA (sgA, sgB, or sgC), and 1.4 µg of human *BMAL1* HDR plasmid (sc-400808-HDR; Santa Cruz Biotechnology, Dallas, TX, USA) using Xfect transfection reagent (Takara Bio USA, San Jose, CA, USA). Puromycin-resistant clones were selected using 1 µg/mL puromycin. *Bmal1* knockout was confirmed by immunoblot analysis using anti-BMAL1 antibody (B-1; Santa Cruz Biotechnology, Dallas, TX, USA) and genomic PCR followed by Sanger sequencing. The following primers were used for genomic PCR and Sanger sequencing: C_fwd, 5' AGATCATCCAATGGCAGAC 3'; C_rev:5' GAGATGACACCCATAGACTTA 3'; B_fwd:5' AAGAAGCTCTTCTGTATGTC 3''; B_rev:5' AATAAGGTCCAAGCTTACCT 3'; A_fwd:5' AAGAGCGATGTCGTTGGAG 3''; A_rev:5' TGCATGGTACAAGTCCTGAAGC 3'. The results of the genomic PCR and Sanger sequencing are summarized in Supplementary Fig. S7. A *Bmal1* knockout clone, named U2OS-P_*Bmal1*_::*Fluc*/Δ*Bmal1*, was used to establish Myc-tagged BMAL1 (MYC-BMAL1) inducible clones.

### Construction of doxycycline-inducible expression plasmid of Myc-BMAL1

The human *BMAL1* open reading frame (ORF) was amplified by PCR using KOD-plus-neo (Toyobo Biotechnology, Osaka, Japan) from the Kazusa Flexi ORF clone FXC03462 (Promega, Madison, WI, USA) and the following primers: Bmal1ORF_fwd, 5' CCGGAATTCATGGCAGACCAGAGAATGGACATTTCT 3'; Bmal1ORF_rev, 5' CGCGGATCCTCACAGCGGCCATGGCAAGTCACTAAAGTC 3'. The PCR product was digested with EcoRI and BamHI and cloned into the EcoRI-BamHI site of pTetOne (Takara Bio USA, San Jose, CA, USA). The Myc-tag was introduced into the resultant plasmid by inverse PCR using KOD-Plus mutagenesis kit (Toyobo Biotechnology, Osaka, Japan). The following primers were used for inverse PCR: Myc-Bmal1_fwd, 5' ACCATGGAGCAGAAGCTGATCTCAGAGGAGGACCTGATGGCAGACCAGAGAATGGACATTTCT 3'; Myc-Bmal1_rev, 5' GAATTCTTTACGAGGGTAGGAAGTGGT 3'. The resulting plasmid was named pTetOne-MycBmal1.

### Establishment of MYC-BMAL1 inducible cell lines

U2OS-P_*Bmal1*_::*Fluc*/Δ*Bmal1* cells were transfected with 5 µg pTetOne-MycBmal1 plasmid and 0.25 µg linear hygromycin marker (Takara Bio USA, San Jose, CA, USA) using Xfect transfection reagent (Takara Bio USA, San Jose, CA, USA). Hygromycin B (300 µg/mL) was added to the cell culture to select positive clones. MYC-BMAL1 protein expression in the isolated clones was evaluated by immunoblot analysis using an anti-Myc-tag mAb (My3, Medical & Biological Laboratories, Tokyo, Japan) in the presence of 1 µg/mL DOX (Takara Bio USA, San Jose, CA, USA). We obtained seven cell lines, which we named U2OS-P_*Bmal1*_::*Fluc*/Δ*Bmal1*/ P_*TRE3Gs*_::*Myc-Bmal1* strains-2, -17, -23, -27, -33, -51, and -59.

### Luminescence measurements

U2OS-P_*Bmal1*_::*Fluc*/Δ*Bmal1*/ P_*TRE3Gs*_::*Myc-Bmal1* cells were plated at a density of 8 × 10^3^ cells/well in 96 well white, clear-bottom culture plates and were cultured for 48 h at 37 °C in 10% CO_2_ to reach confluence. The cells were treated with DOX (0–10 µg/mL) and incubated for another 48 h. The medium was then changed to medium for luminescence measurement^35^ supplemented with the same DOX concentration. Luminescence was measured every 20 min for approximately 1 week using a plate reader (Enspire; PerkinElmer, Waltham, MA, USA). An integration time of 1 s was employed for each measurement.

For dual-reporter measurements, U2OS-P_*Bmal1*_::*Fluc*/Δ*Bmal1*/P_*TRE3Gs*_::*Myc-Bmal1* strain-2 cells were plated in 35 mm culture dishes (Nunc EasYDish; Thermo Fisher Scientific, Waltham, MA, USA) at a density of 2 × 10^5^ cells/dish in the presence of 0, 0.01, 0.1, and 1 µg/mL DOX. The next day, cells were transiently transfected with 2.5 µg pP_*Per2*_::*Eluc* plasmid containing a 423-bp fragment of the m*Per2* promoter region^36^ inserted at the BglII-EcoRI site of the pEluc(PEST)-test (Toyobo Biotechnology, Osaka, Japan) using Xfect transfection reagent (Takara Bio USA, San Jose, CA). Then, the cells were incubated for another 24 h. The medium was changed to luminescence measurement medium supplemented with DOX at the same concentration. Luminescence from P_*Bmal1*_::*Fluc* and P_*Per2*_::*Eluc* reporters was measured simultaneously using a Kronos-Dio instrument (ATTO, Tokyo, Japan) equipped with a 620 nm long pass filter for 6 days according to the method described by Ono et al.^22^.

### Immunoblot analysis of circadian clock proteins

U2OS-P_*Bmal1*_::*Fluc*/ΔBmal1/ P_*TRE3Gs*_::*Myc-Bmal1* strain-2 cells were plated at a density of 2 × 10^5^ cells/dish in 35 mm dishes and cultured at 37 °C with 10% CO_2_. When cells reached confluence, 0 to 10 µg/mL DOX was added and the cells were cultured for another 48 h. The medium was changed to DMEM supplemented with 2% B-27 (Gibco), 2 mM glutamine, 100 U/mL penicillin, 100 µg/mL streptomycin, 100 nM dexamethasone, and DOX at the same concentration. The cells were lysed with 1 × SDS sample buffer 0–52 h after adding dexamethasone. Samples were sonicated using a Bioruptor UCW 310 (Cosmo Bio, Tokyo, Japan) for 25 cycles of 30 s sonication at 310 W, followed by 30 s of rest in ice water. The samples were centrifuged at 20,000 × *g* for 10 min at 4 °C to remove debris and denatured at 95 °C for 5 min. Protein concentration was measured using Pierce 660 nm Protein Assay Reagent (Thermo Fisher Scientific, Waltham, MA, USA). Samples were separated by SDS-PAGE on 7.5% gels (E-R7.5L, ATTO, Tokyo, Japan) loaded at 20 µg protein/lane. The bands were transferred to polyvinylidene difluoride (PVDF) membranes using an iBlot 2 Dry Blotting system (Thermo Fisher Scientific, Waltham, MA, USA). The membranes were stained with Ez Stain Aqua Mem solution (ATTO, Tokyo, Japan) to measure the total protein levels. Images were captured using a LuminoGraph II EM instrument (ATTO, Tokyo, Japan) in bright-field mode. The membranes were destained and blocked overnight with 5% skim milk dissolved in Tris-buffered saline containing 0.2% Tween 20 (TBS-T) at 4 °C. The membranes were incubated with primary antibody diluted in 5% skim milk in TBS-T for 1.5 h at room temperature and washed three times with TBS-T for 10 min. The membranes were incubated with secondary antibody for 1.5 h at room temperature and were washed as described above. For luminescence detection, the membranes were treated with ECL prime reagent (Cytiva, Marlborough, MA, USA). Signals were captured using a LuminoGraph II EM instrument (ATTO, Tokyo, Japan). Quantification of band intensity was performed using ImageJ software (NIH, USA). The background signal was measured in a signal-free area of the membrane and subtracted from the intensity of each band, which was then normalized to the total protein.

The antibodies used and their dilutions were as follows: anti-Myc-tag mAb (My3; MBL, Tokyo, Japan), 1:200; anti-NR1D1 pAb (Rev-Erbα) (PM092; MBL, Tokyo, Japan), 1:200; anti-CLOCK (18,094–1-AP; Proteintech, Chicago, IL, USA), 1:500; anti-ARNTL (BMAL1) (14,268–1-AP; Proteintech, Chicago, IL, USA), 1:3000; HRP-linked anti-mouse IgG (NA931; Cytiva, Marlborough, MA, USA), 1:1000; and HRP-linked anti-rabbit IgG (NA934; Cytiva), 1:1000.

### Quantitative reverse transcription PCR (RT-PCR)

Samples were collected from 24 to 52 h after the addition of dexamethasone and were prepared as described above. Total RNA was extracted using RNeasy-plus Micro kit (QIAGEN, Venlo, Netherlands). The RNA concentration was measured using a spectrophotometer. Reverse transcription was performed using ReverTra Ace (Toyobo Biotechnology, Osaka, Japan) and 3 µg total RNA. Quantitative RT-PCR was performed using QuantStudio 3 (Applied Biosystems, Waltham, MA, USA) in 20 µL reactions containing 10 µL 2 × TaqMan gene expression master mix (Applied Biosystems), 4 µL reverse transcription reaction mixture, and 1 µL 20 × TaqMan gene expression assay (HS01587195_m1 for *BMAL1* and HS02786624_g1 for *GAPDH*, Applied Biosystems). Relative expression was calculated using Pfaffl’s method^37^, with *GAPDH* used as an internal control.

### Statistical analysis

Significant differences between more than two groups was evaluated using analysis of variance (ANOVA) followed by Tukey’s multiple comparison test using GraphPad Prism version 7.0 (GraphPad Software, San Diego, CA, USA). Rhythmicity was determined by the JTK cycle test^23^. All *P*-values were from two-tailed tests. *P* < 0.05 was considered statistically significant.

## Supplementary Information

Supplementary Information.

## Data Availability

All data generated or analyzed during this study are included in this published article (and its supplementary information files).
