# Supplementary material for: Level of constitutively expressed BMAL1 affects the robustness of circadian oscillations
Source: Sci Rep. 2022 Nov 14;12:19519. doi: 10.1038/s41598-022-24188-4 (PMC9663716; doi:10.1038/s41598-022-24188-4)

Supplementary Information

Level of constitutively expressed BMAL1 affects the robustness of circadian oscillations

Apirada Padlom[a], Daisuke Ono[b,c], Rio Hamashima[a], Yuko Furukawa[d], Takashi Yoshimura[a,d], Taeko Nishiwaki-Ohkawa[a,d,*]

[a]Laboratory of Animal Integrative Physiology, Graduate School of Bioagricultural Sciences, Nagoya University, Furo-cho, Chikusa-ku, Nagoya 464-8601, Japan; [b]Department of Neuroscience II, Research Institute of Environmental Medicine, Nagoya University, Nagoya 464-8601, Japan; [c]Department of Neural Regulation, Nagoya University Graduate School of Medicine, Nagoya 464-8601, Japan; [d]Institute of Transformative Bio-Molecules (WPI-ITbM), Nagoya University, Furo-cho, Chikusa-ku, Nagoya 464-8601, Japan.

*Taeko Nishiwaki-Ohkawa

Email: tohkawa@agr.nagoya-u.ac.jp

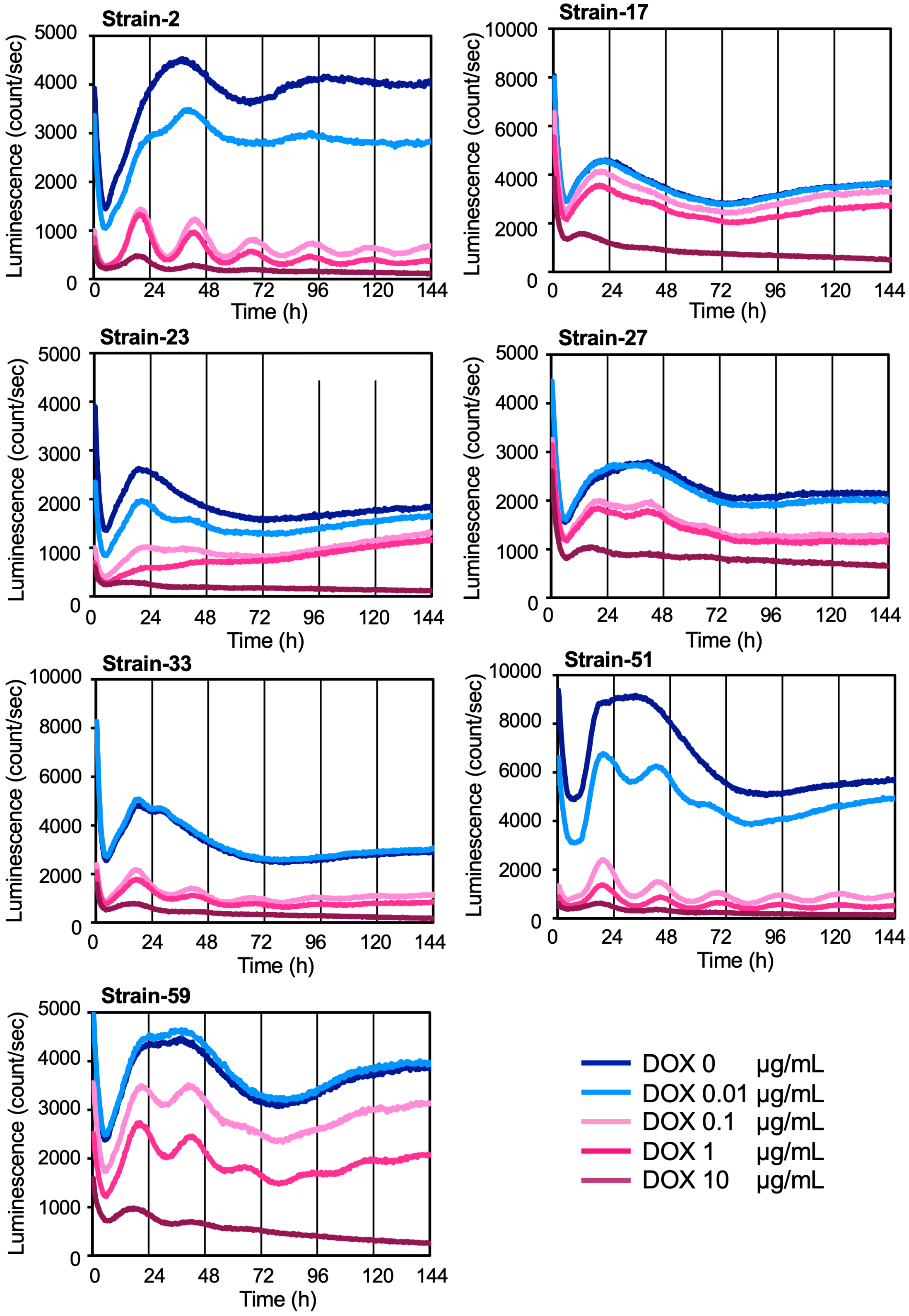
Strain-2
Strain-17
Strain-23
Strain-27
Strain-33
Strain-51
Strain-59
Luminescence (count/sec)
Time (h)
0
24
48
72
96
120
144
0
1000
2000
3000
4000
5000
6000
8000
10000
DOX 0 μg/mL
DOX 0.01 μg/mL
DOX 0.1 μg/mL
DOX 1 μg/mL
DOX 10 μg/mL

Supplementary Figure S1

U2OS-P_{Bmal1}::*Fluc*/Δ*Bmal1*/P_{TRE3Gs}::*Myc-Bmal1* strains -2, -17, -23, -27, -33, -51, and -59 were subjected to P_{Bmal1}::*Fluc* reporter luminescence measurements. Doxycycline (DOX) treatment was initiated 48 h before starting measurements. The average of the triplicate measurements is presented. The DOX concentrations are indicated on the bottom-right side of the figure.

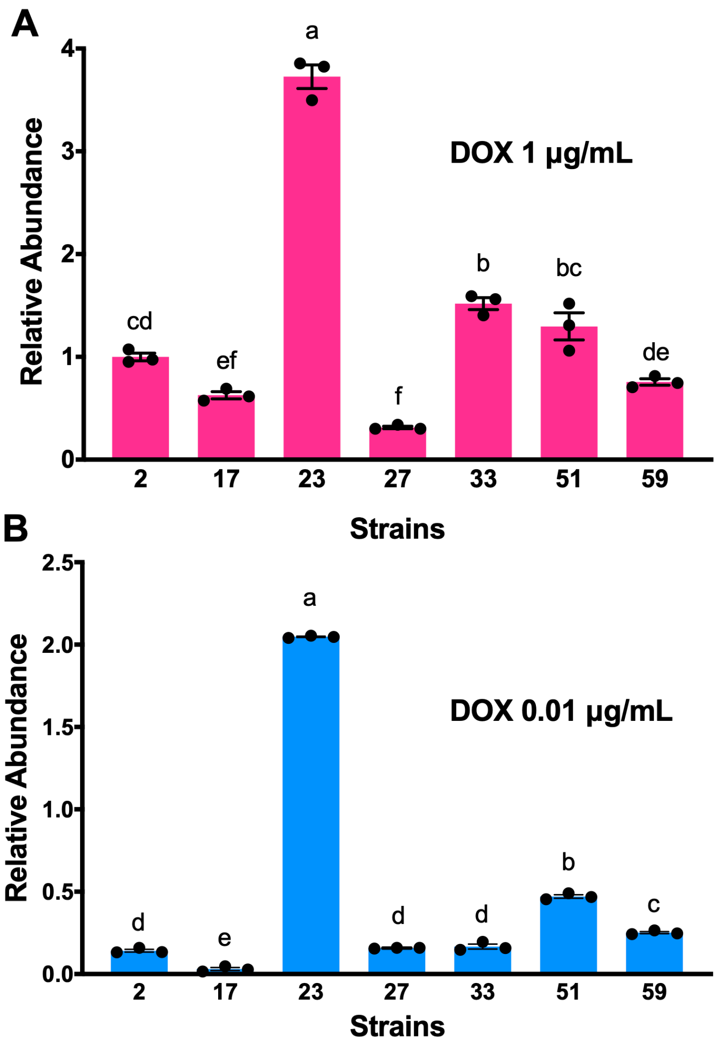

Supplementary Figure S2

Total protein samples were collected from seven U2OS-P_{Bmal1}::*Fluc*/Δ*Bmal1*/P_{TRE3Gs}::*Myc-Bmal1* strains 48 h after adding 1 (A) or 0.01 µg/mL DOX (B). Data are normalized by the average values from strain-2 treated with 1 µg/mL DOX and are shown as the relative value. The results were shown as mean ± SEM. N = 3 samples/group, one-way ANOVA followed by Tukey's multiple comparison test. Different characters (a, b, c, d, e) indicate significant differences ($P < 0.05$).

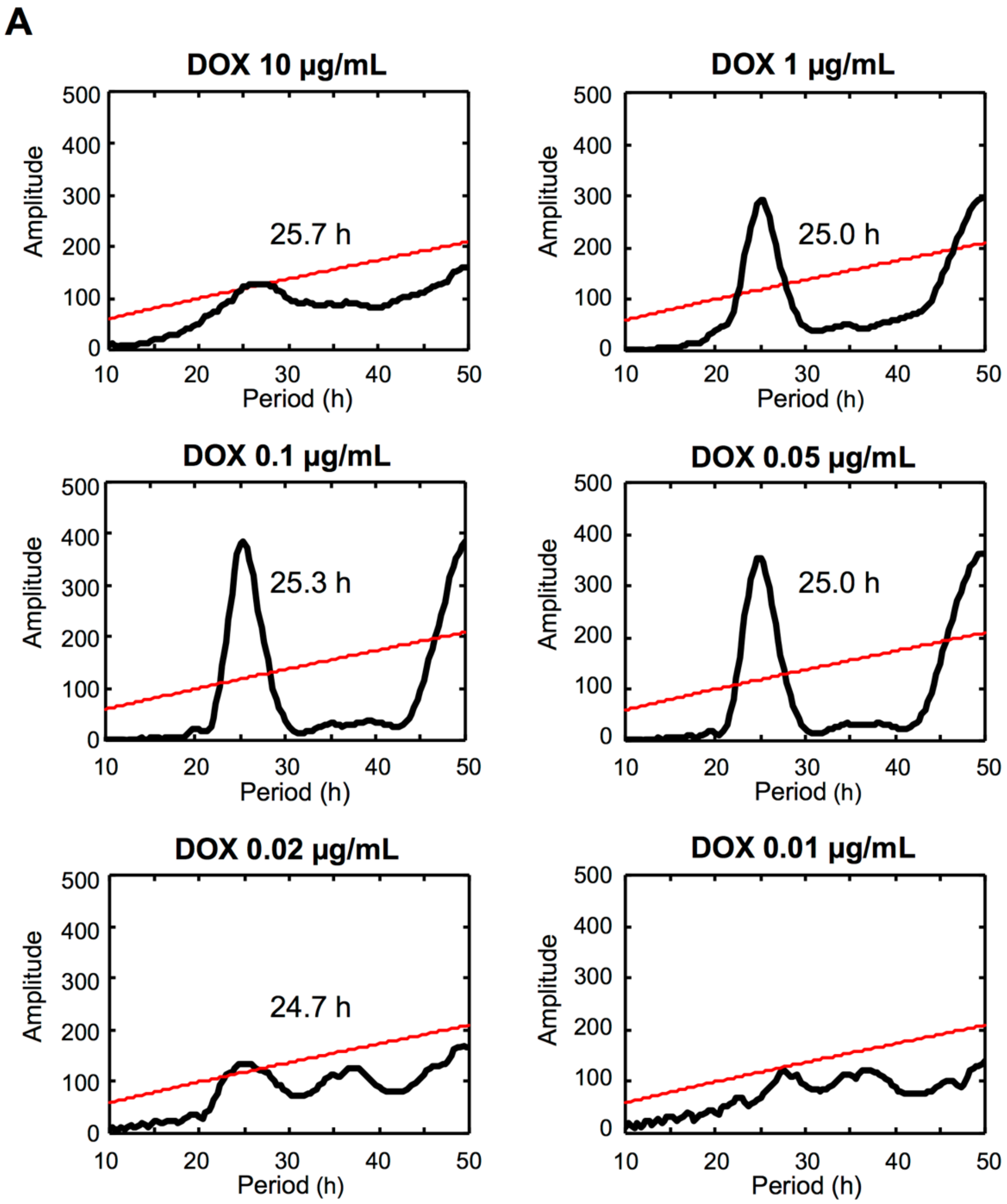
A
DOX 10 μg/mL
25.7 h
DOX 1 μg/mL
25.0 h
DOX 0.1 μg/mL
25.3 h
DOX 0.05 μg/mL
25.0 h
DOX 0.02 μg/mL
24.7 h
DOX 0.01 μg/mL
Amplitude
Period (h)
0
100
200
300
400
500
10
20
30
40
50

B

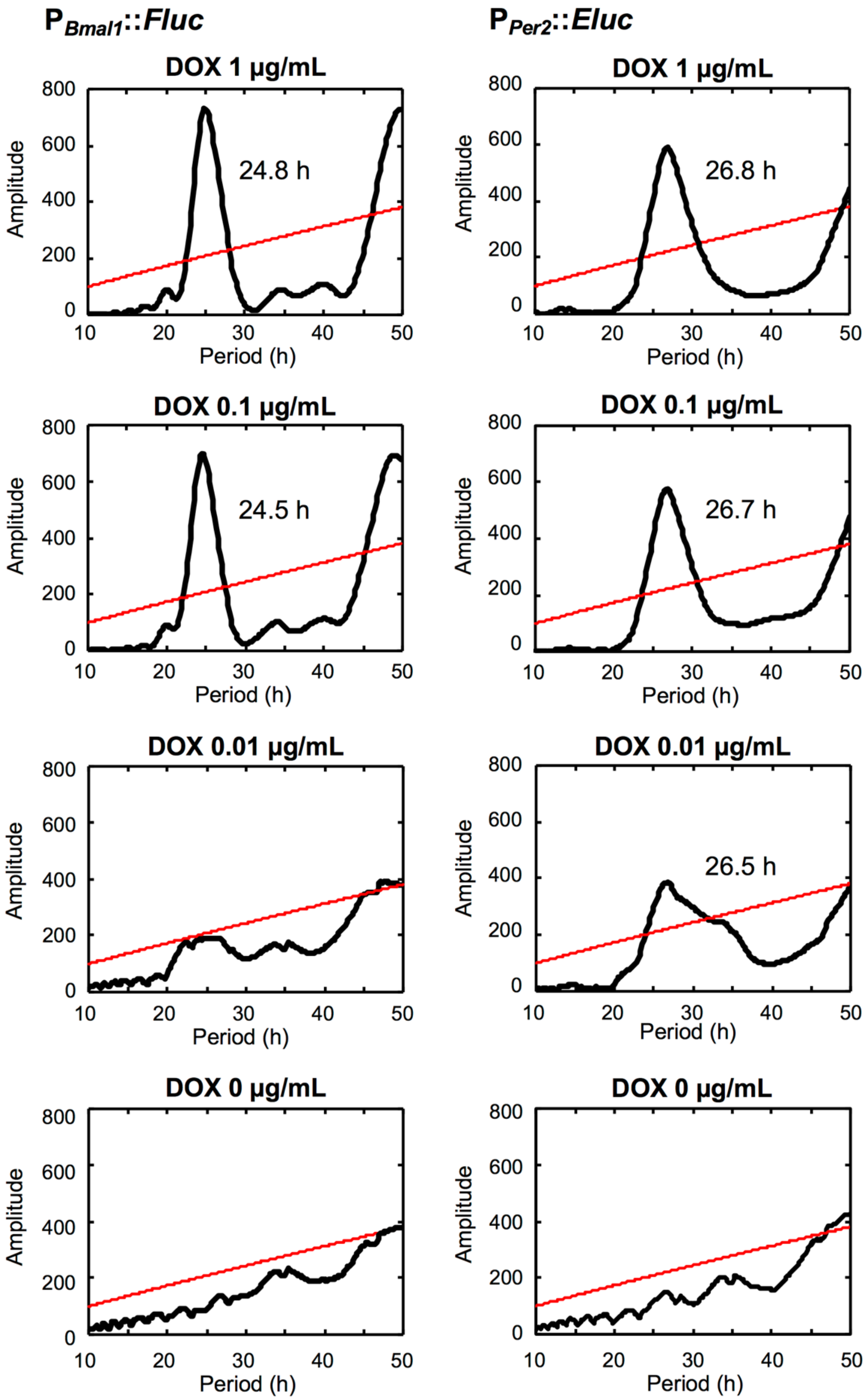

P_{Bmal1}::*Fluc*
P_{Per2}::*Eluc*
DOX 1 µg/mL
24.8 h
26.8 h
DOX 0.1 µg/mL
24.5 h
26.7 h
DOX 0.01 µg/mL
26.5 h
DOX 0 µg/mL
Amplitude
Period (h)

Supplementary Figure S3

Luminescence traces from 0 to 144 h shown in Fig. 1B (A) and 0 to 141 h shown in Fig. 2 (B) were subjected to chi-square periodogram analysis using Lumicycle analysis software (ActiMetrics; Wilmette, IL, USA) to assess the period of their oscillatory component and its significance. The red lines in the periodograms indicate *P*=0.05.

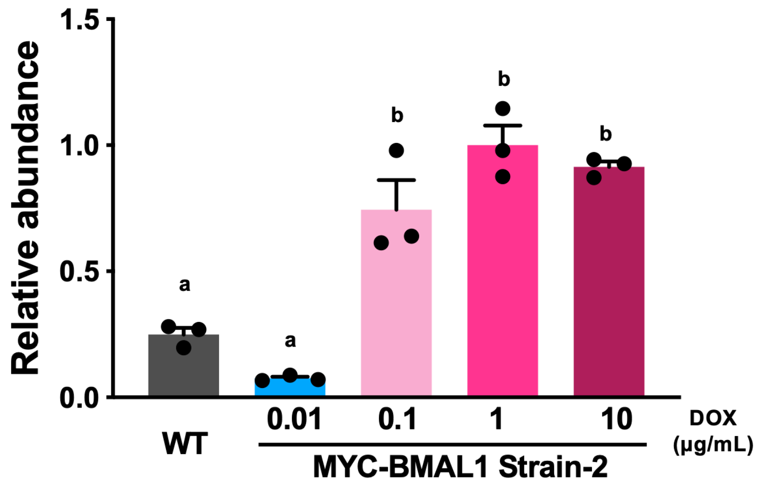

Supplementary Figure S4

Total protein samples were collected from U2OS-P_{Bmal1}::*Fluc* (WT) and U2OS-P_{Bmal1}::*Fluc*/Δ*Bmal1*/P_{TRE3Gs}::*Myc-Bmal1* strain-2 (MYC-BMAL1 Strain-2) cells every 4 h from 0 to 52 h after adding 100 nM dexamethasone. Equal amounts of the protein samples collected within each group were mixed and subjected to immunoblot analysis using anti-BMAL1 antibody. The graph shows the relative abundance of endogenous BMAL1 protein or MYC-BMAL1 protein after treatment with different concentrations of doxycycline (DOX). Results were normalized using the average value of MYC-BMAL1 at 1 µg/mL DOX. The data are shown as the mean ± SEM. N = 3 samples/group, one-way ANOVA followed by Tukey's multiple comparison test. Different characters (a, b) indicate significant differences ($P < 0.05$).

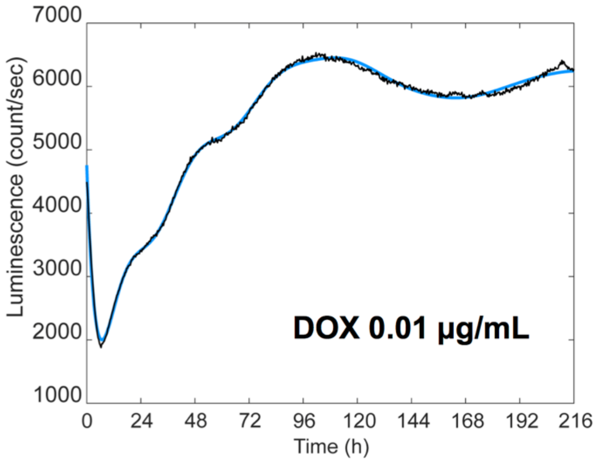

Supplementary Figure S5

Transient response analysis of luminescence data of P_{Bmal1} activity in the presence of 0.01 µg/mL doxycycline (DOX). P_{Bmal1} activity was approximated using the following six-order transfer function with six poles and six zeros.

$$G(s) = \frac{4756s^6+431.4s^5+276s^4+14.22s^3+3.075s^2+0.04085s+0.006588}{s^6+0.3191s^5+0.06675s^4+0.01035s^3+0.0006645s^2+3.197e^{-5}s+1.083e^{-6}}$$

The measured luminescence intensity is shown in black and the simulated data are shown in blue.

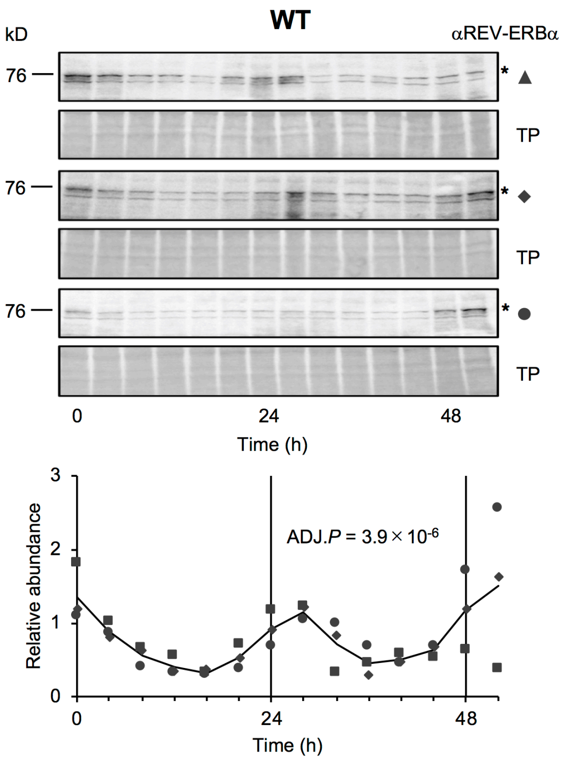

Supplementary Figure S6

Time course of REV-ERBα protein expression in WT U2OS. Images showing the protein bands detected for REV-ERBα and total protein (TP) stains (upper panels). Asterisks indicate nonspecific bands. Markers (▲,◆, and ●) indicate three biological replicates. The protein amount was quantified using densitometry (lower panel). The relative expression of REV-ERBα protein was calculated by the density of each band vs. total protein and was normalized against the intensity of pooled 0 to 52 h samples. Black lines indicate the average values of three biological replicates. Significant rhythmicity was detected for REV-ERBα in WT U2OS (JTK cycle test, ADJ.*P* =3.9 $\times 10^{-6}$)

A

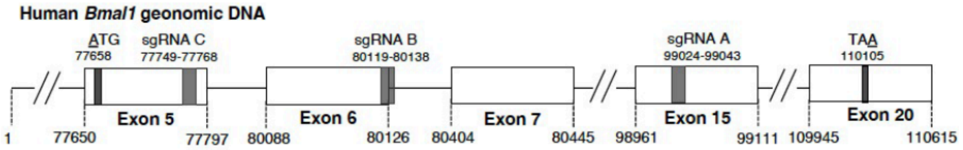

B

Primers		Amplicon size (bp)		
Forward primer	Reverse primer	Predicted	ΔBmal1	
C_fwd	C_rev	496	No Amp	
C_fwd	B_rev	2816	446	➡ Amplicon BC
C_fwd	A_rev	21663	361	➡ Amplicon AC

C

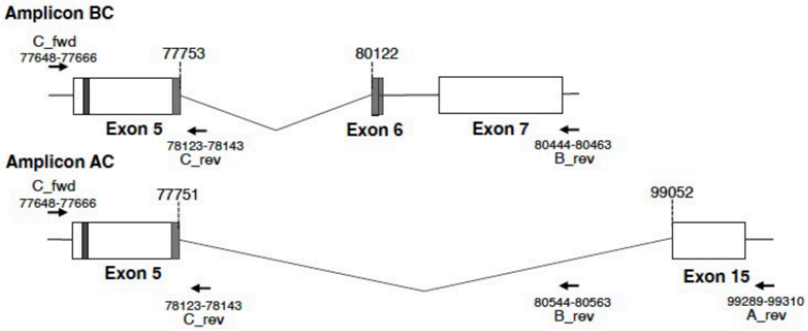

D

Amplicon BC

AGATCATCCAATGGCAGACCAGAGAATGGACATTTCTTCAACCATCAGTGATTTCATGTCCCCGGGCCCC
ACCGACCTGCTTTCCAGCTCTCTTGGTACCAGTGGGAAGGTACCATGAACCTAGTAATTTGAACTTCAGC
ATCCTTATAGCCATTTTCTTTGCACTGTTACACATTCTGTTACTTGGGGCAGCACCCATGTCCTCAACTG
GAGATGAGCAAGGAGGCCGTGAGCCTGTGGGCGCTCACTGTGTCCCTCCAACCCCCAGTCCCCTTGTGTG
TCTGCAGAGAGATGACAGGATCAGGCAGAAGAAAACAGCAATGTGTAACTTTGCCATTCATCTCCAGAGA
ATTATGTTTTTATCTTTTGCTTTTTCCTCCCCCCAGGTTAGAATATACAGAACACCAAGGAAGGATAAAA
AATGCAAGGTAAGCTTGGACCTTATT

E

Amplicon AC

AGATCATCCAATGGCAGACCAGAGAATGGACATTTCTTCAACCATCAGTGATTTCATGTCCCCGGGCCCC
ACCGACCTGCTTTCCAGCTCTCTTGGTACCAGTTTCATGAACCCTTGGACCAAGGAAGTAGAATATATTG
TCTCAACTAACACTGTTGTTTTGTAAGTACTTTTCCTATATCTGAAGCTCCCCTTGCTTCAAACAGATGC
CTAGGGTTCCTCATCTGGGAAATGGGGTGCAGGCAACATCCAGTATCACATCCTTTAATGCCATCTTGCT
AATACCTGTGAAGCCTCAGGACTGGCAGAAGCTAGAAAGGATTGTTAACAAAGGGACAAGCTTCAGGACT
TGTACCATGCA

Supplementary Figure S7

(A) Schematic diagram of the human *BMAL1* gene. The DNA sequence of the human ARNTL gene (ID 408), a synonym of *BMAL1*, was obtained from the GenBank database. The positions of the sgRNAs and the start and stop codons are shown in the figure. (B) Results of genomic PCR analysis of the regions flanking the sgRNA-binding sites. Genomic DNA was prepared from U2OS-P$_{Bmal1}$::*Fluc*/Δ*Bmal1* cells and was subjected to PCR using the indicated primers. Amplicon sizes predicted from the database (predicted) and those obtained from the experiment (*ΔBmal1*) are shown. No amplification was observed using C_fwd and C_rev primers. (C) Schematic diagram of amplicons BC and AC. The primer positions are shown in panels C-E. Nucleotides 77753 to 80112 and 77751 to 99052 were deleted in amplicons BC and AC, respectively. (D, E) DNA sequences of amplicons BC (D) and AC (E) were determined by direct Sanger sequencing of the amplicons.

Supplementary Figure S8

Gels and blots

After blotting, the membranes were stained with Ez Stain Aqua Mem solution (ATTO, Tokyo, Japan) to measure total protein levels. Images were captured using a LuminoGraph II EM instrument (ATTO, Tokyo, Japan) in bright field mode. The membranes were cut and destained before reacting with antibodies. The ECL Rainbow Marker Full Range (RPN800E, Cytiva, Marlborough, MA, USA) was used as the molecular weight marker. To detect Myc-tagged BMAL1 (MYC-BMAL1) bands (Fig. 3, A-C and Fig. S2) and endogenous BMAL1 bands (Fig. S4), we cut the membranes above the 102 kD marker at the upper side and below the 52 kD markers at the lower side, and reacted them with mouse anti-Myc-tag mAb and rabbit anti-BMAL1 antibody. REV-ERBα bands were detected using a rabbit anti-REV-ERBα antibody (Fig.4, A-C). The same blots were re-probed after erasing the MYC-BMAL1 signals by treatment with 15% H_2O_2 solution. For detection of CLOCK bands (Fig. 4, D-E), membranes were cut above the 150 kD marker on the upper side and below the 76 kD marker on the lower side. The bands were detected using ECL Prime reagent. Images were acquired using a LuminoGraph II EM instrument equipped with ImageSaver 7 software. The auto-exposure settings were × 5 sensitivity and 1 × 1 binning. Total protein staining and immunoblotting data were quantified using ImageJ software (NIH, USA). Prior to quantification, we confirmed that the signals were unsaturated. Images of chemiluminescence and pre-stained molecular weight markers were merged automatically using Luminograph II EM. We used merged images only to confirm the positions of molecular weight markers. In Figs 3A, 4A, S2, and S4, we only presented the graphs showing the relative amount of the proteins.

Fig 3A Anti- Anti-Myc (DOX 0-10 μg/mL)

Total protein

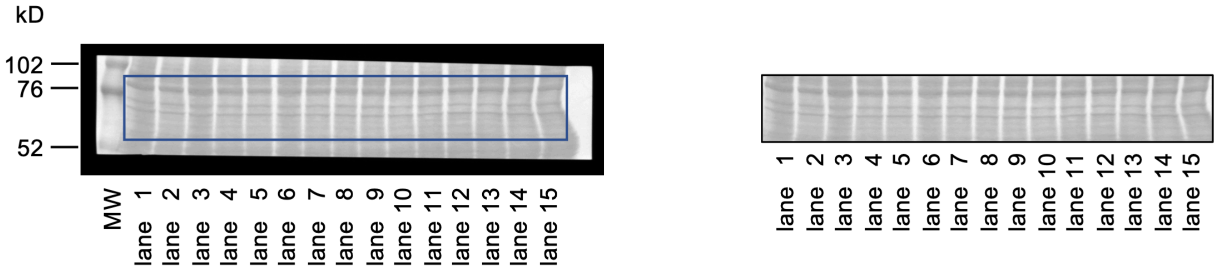

Chemiluminescence

Biological replicate 1

Original

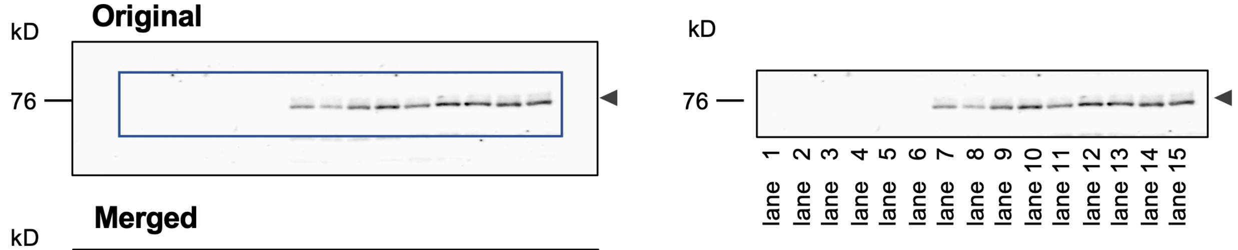

Merged

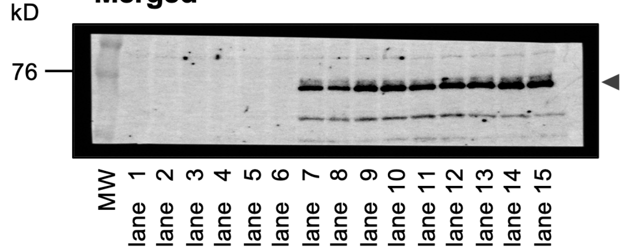

lane 1 : Myc-Bmal1 strain-2 DOX 0 μg/mL Biological replicate 1
lane 2 : Myc-Bmal1 strain-2 DOX 0 μg/mL Biological replicate 2
lane 3 : Myc-Bmal1 strain-2 DOX 0 μg/mL Biological replicate 3
lane 4 : Myc-Bmal1 strain-2 DOX 0.01 μg/mL Biological replicate 1
lane 5 : Myc-Bmal1 strain-2 DOX 0.01 μg/mL Biological replicate 2
lane 6 : Myc-Bmal1 strain-2 DOX 0.01 μg/mL Biological replicate 3
lane 7 : Myc-Bmal1 strain-2 DOX 0.1 μg/mL Biological replicate 1
lane 8 : Myc-Bmal1 strain-2 DOX 0.1 μg/mL Biological replicate 2
lane 9 : Myc-Bmal1 strain-2 DOX 0.1 μg/mL Biological replicate 3
lane 10 : Myc-Bmal1 strain-2 DOX 1 μg/mL Biological replicate 1
lane 11 : Myc-Bmal1 strain-2 DOX 1 μg/mL Biological replicate 2
lane 12 : Myc-Bmal1 strain-2 DOX 1 μg/mL Biological replicate 3
lane 13 : Myc-Bmal1 strain-2 DOX 10 μg/mL Biological replicate 1
lane 14 : Myc-Bmal1 strain-2 DOX 10 μg/mL Biological replicate 2
lane 15 : Myc-Bmal1 strain-2 DOX 10 μg/mL Biological replicate 3

Fig 3B Anti-Myc DOX 0.1 μg/mL

Total protein

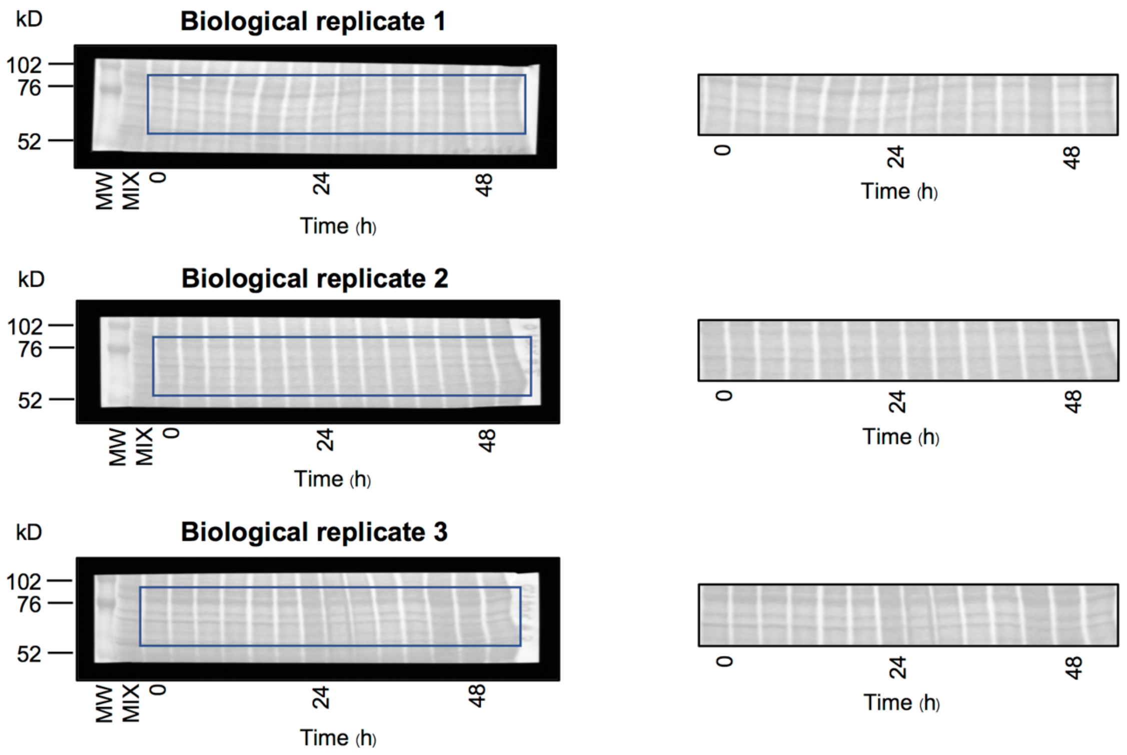

Chemiluminescence

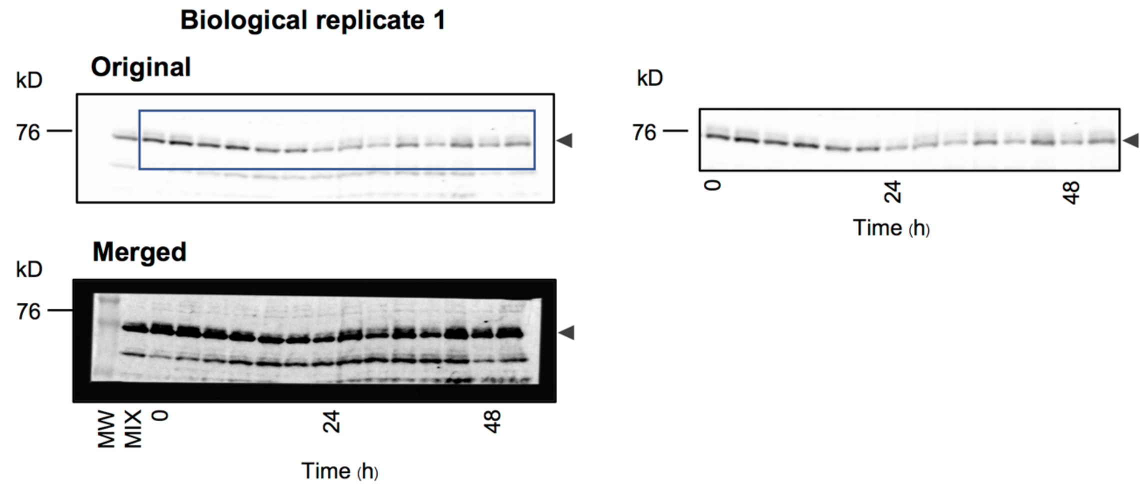

Figure continued on next page

Fig 3B Anti-Myc DOX 0.1 μg/mL (cont.)

Chemiluminescence

Biological replicate 2

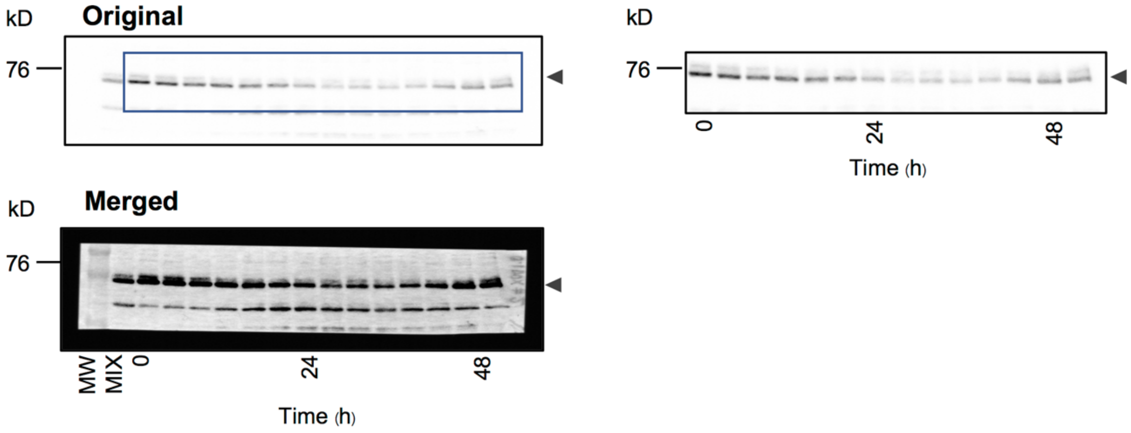

Biological replicate 3

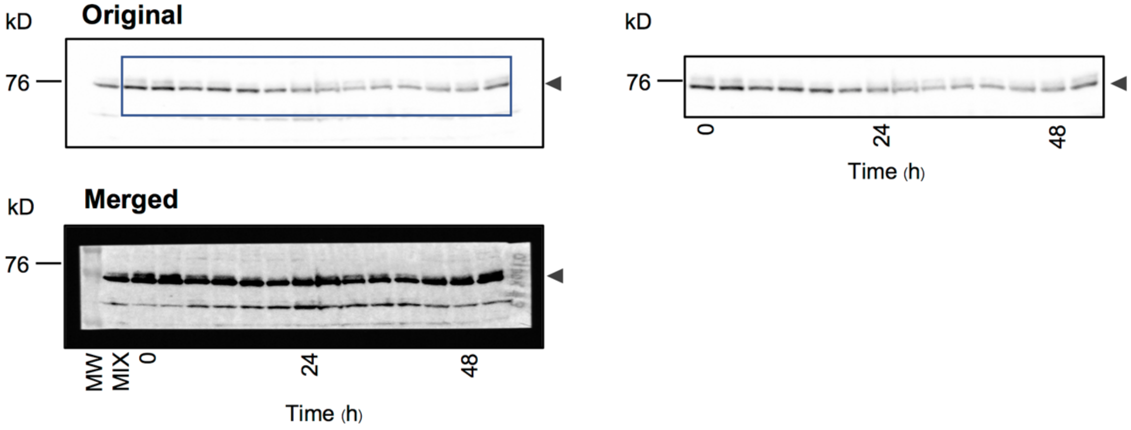

Fig 3C Anti-Myc DOX 1 µg/mL

Total protein

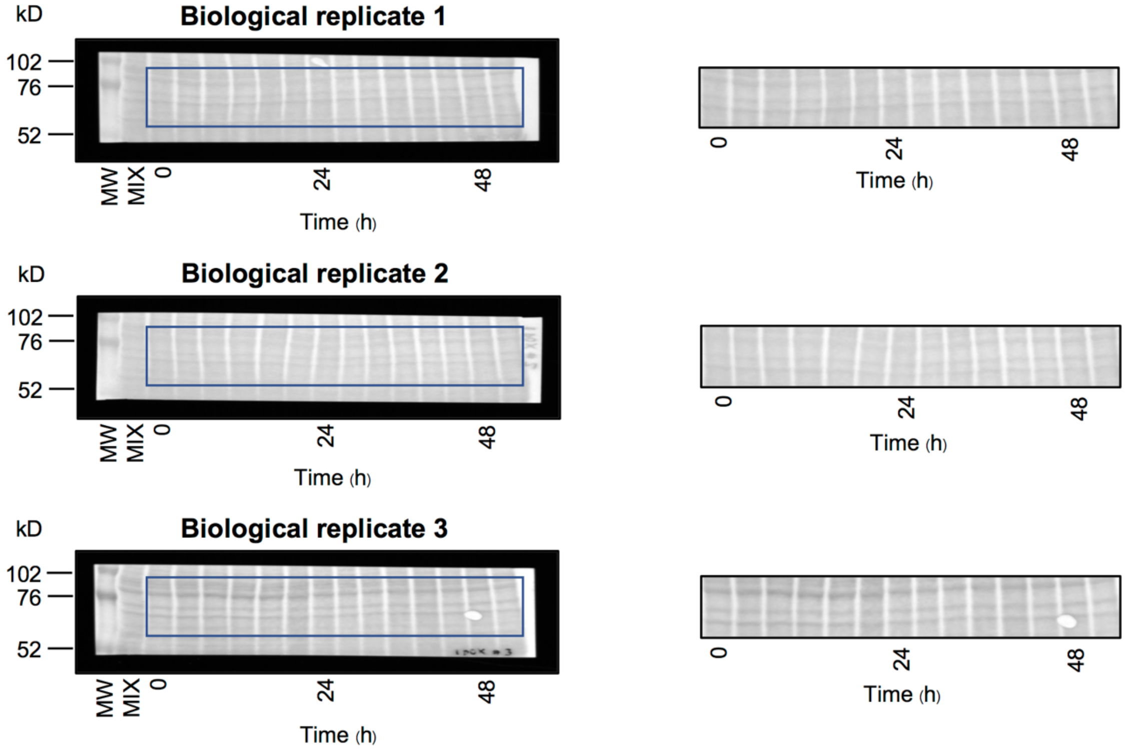

Chemiluminescence

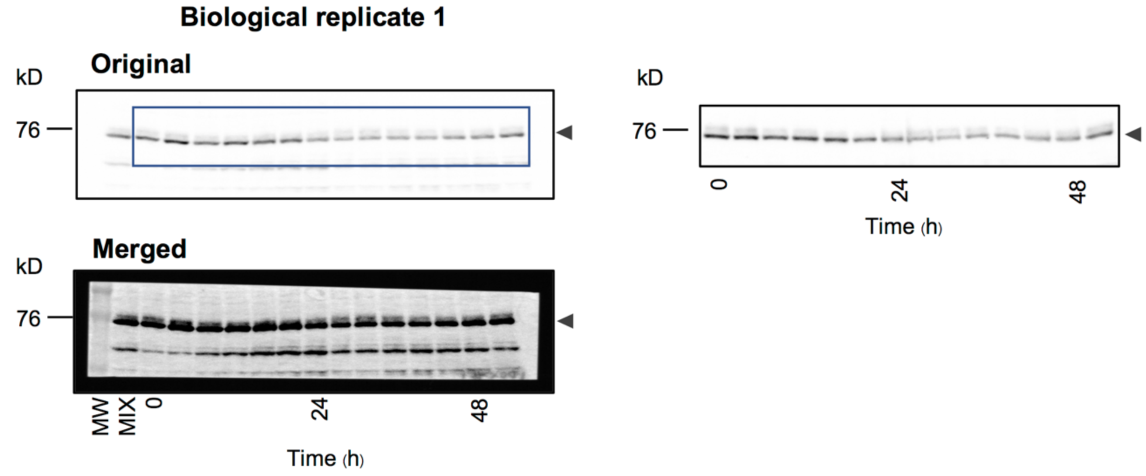

Figure continued on next page

Fig 3C Anti-Myc DOX 1 μg/mL (cont.)

Chemiluminescence

Biological replicate 2

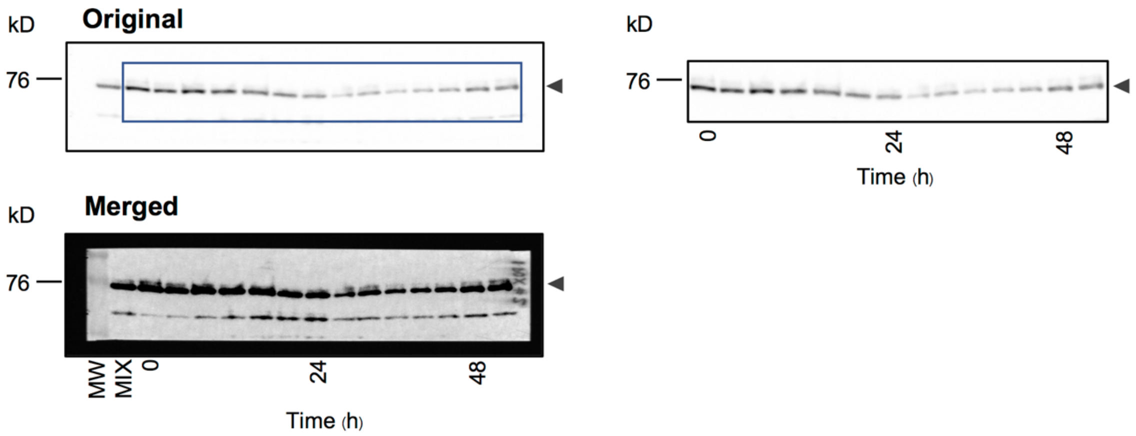

Biological replicate 3

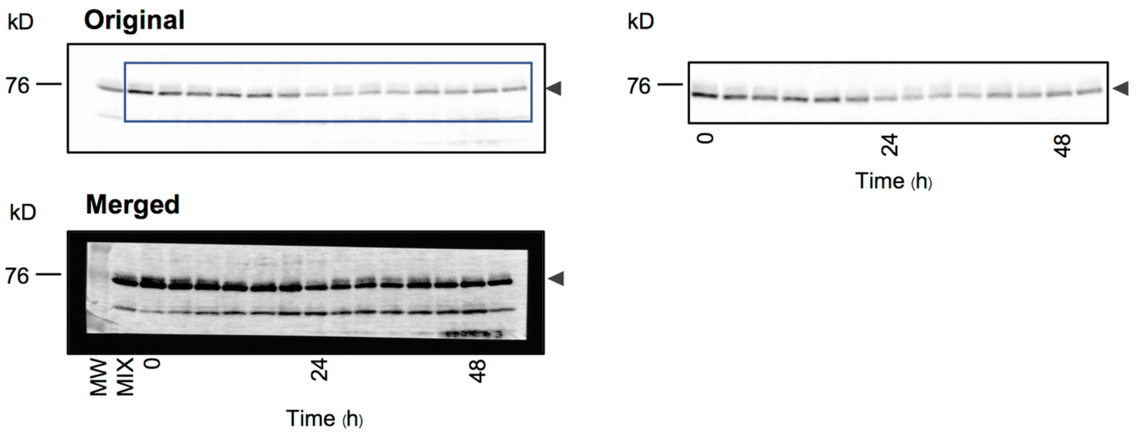

Fig 4A Anti-REV-ERBα (DOX 0-10 μg/mL)

Total protein

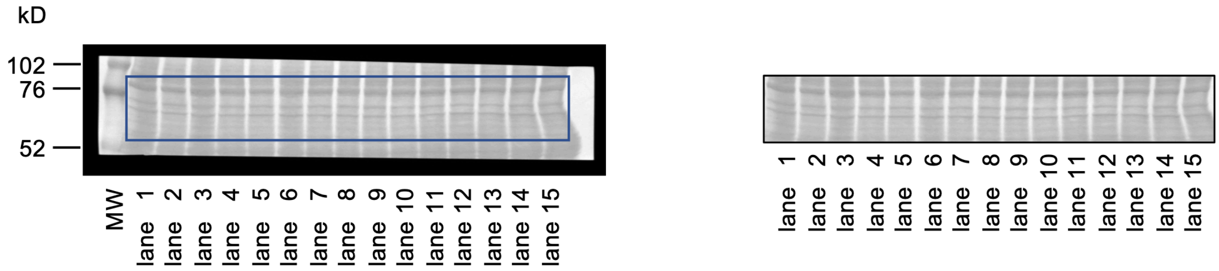

Chemiluminescence

Biological replicate 1

Original

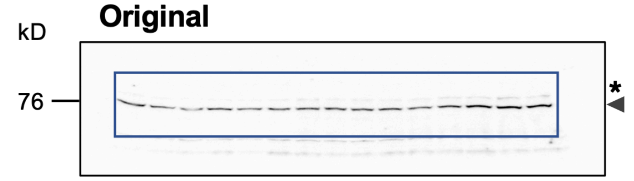

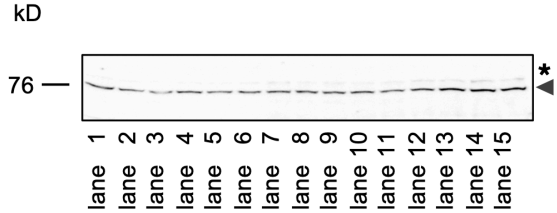

Merged

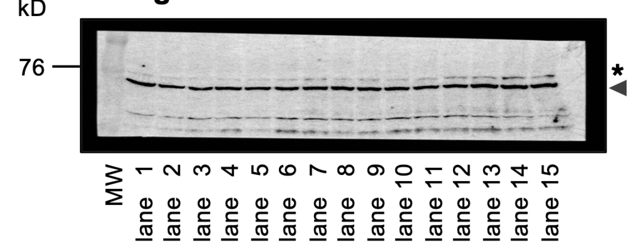

lane 1 : Myc-Bmal1 strain-2 DOX 0 μg/mL Biological replicate 1
lane 2 : Myc-Bmal1 strain-2 DOX 0 μg/mL Biological replicate 2
lane 3 : Myc-Bmal1 strain-2 DOX 0 μg/mL Biological replicate 3
lane 4 : Myc-Bmal1 strain-2 DOX 0.01 μg/mL Biological replicate 1
lane 5 : Myc-Bmal1 strain-2 DOX 0.01 μg/mL Biological replicate 2
lane 6 : Myc-Bmal1 strain-2 DOX 0.01 μg/mL Biological replicate 3
lane 7 : Myc-Bmal1 strain-2 DOX 0.1 μg/mL Biological replicate 1
lane 8 : Myc-Bmal1 strain-2 DOX 0.1 μg/mL Biological replicate 2
lane 9 : Myc-Bmal1 strain-2 DOX 0.1 μg/mL Biological replicate 3
lane 10 : Myc-Bmal1 strain-2 DOX 1 μg/mL Biological replicate 1
lane 11 : Myc-Bmal1 strain-2 DOX 1 μg/mL Biological replicate 2
lane 12 : Myc-Bmal1 strain-2 DOX 1 μg/mL Biological replicate 3
lane 13 : Myc-Bmal1 strain-2 DOX 10 μg/mL Biological replicate 1
lane 14 : Myc-Bmal1 strain-2 DOX 10 μg/mL Biological replicate 2
lane 15 : Myc-Bmal1 strain-2 DOX 10 μg/mL Biological replicate 3

Fig 4B Anti-REV-ERBα DOX 0.1 μg/mL

Total protein

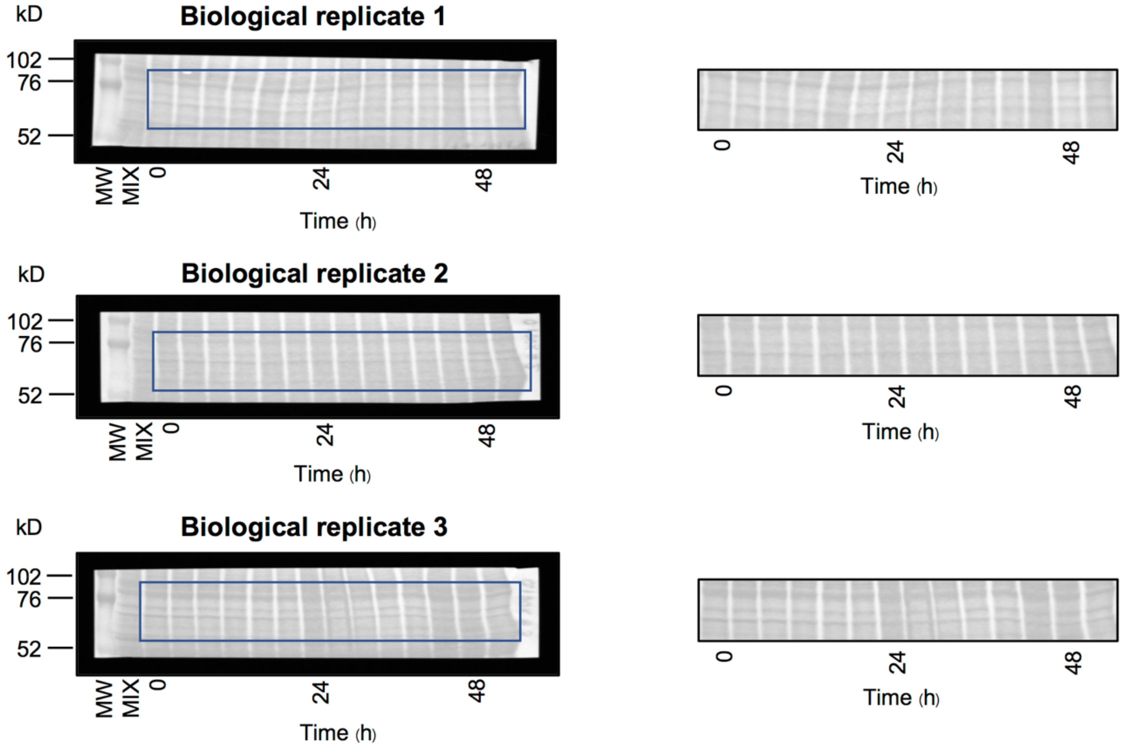

Chemiluminescence

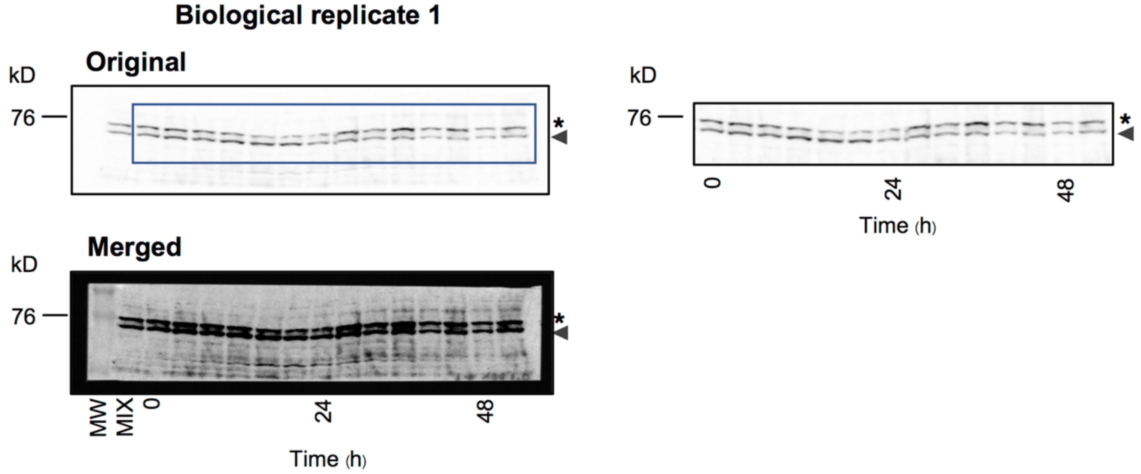

Figure continued on next page

Fig 4B Anti-REV-ERBα DOX 0.1 μg/mL (cont.)

Chemiluminescence

Biological replicate 2

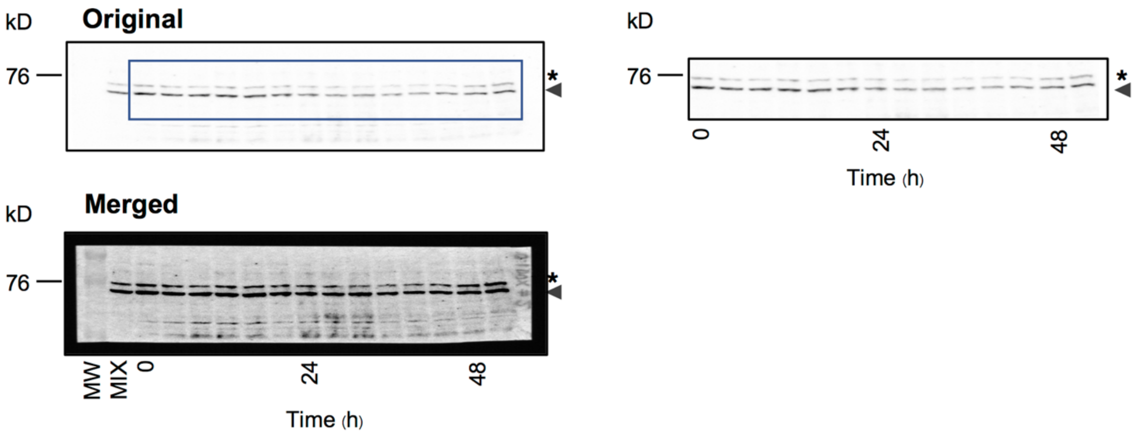

Biological replicate 3

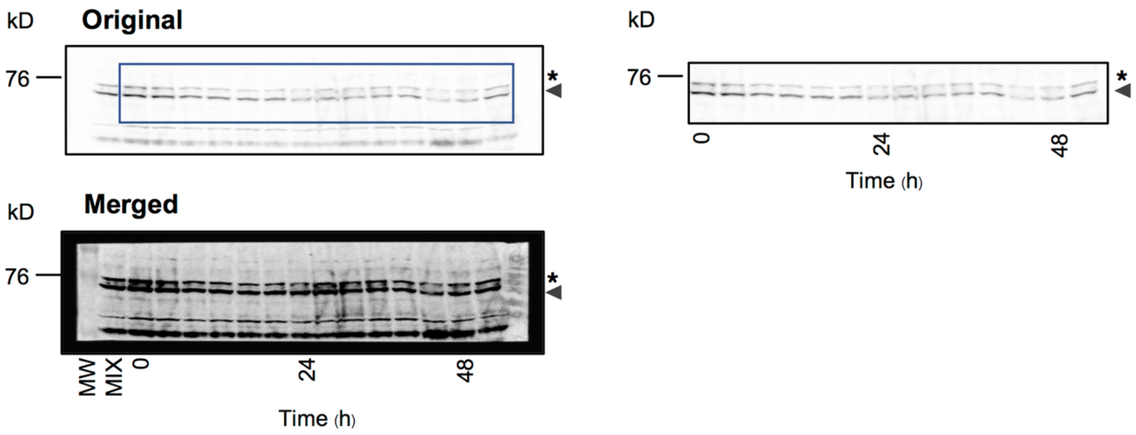

Fig 4C Anti-REV-ERBα DOX 1 μg/mL

Total protein

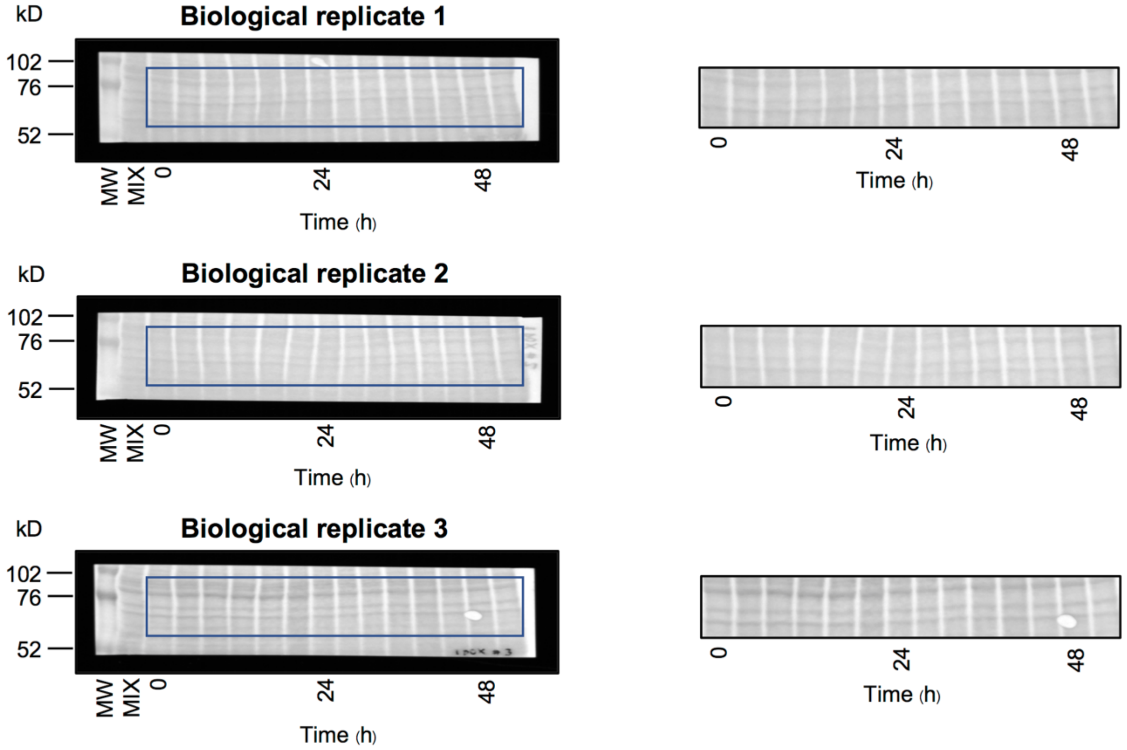

Chemiluminescence

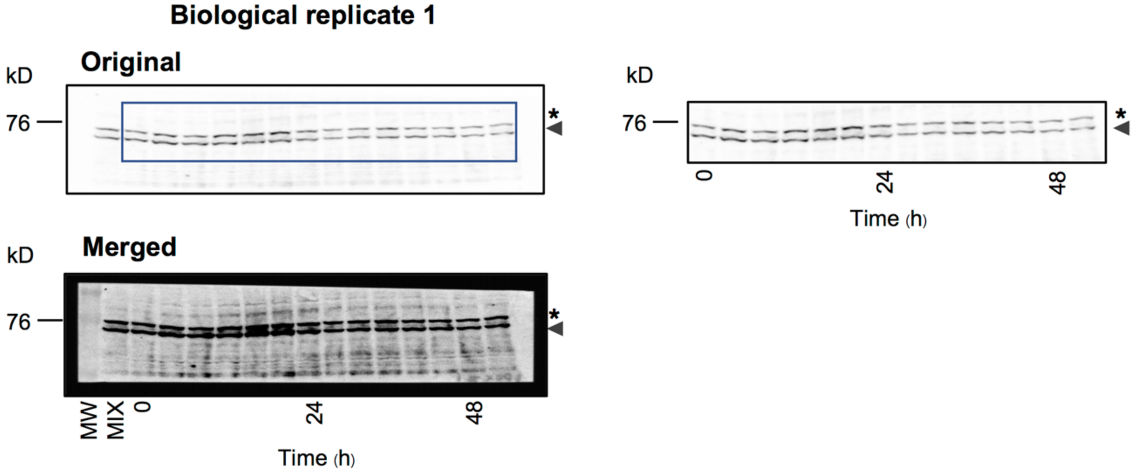

Figure continued on next page

Fig 4C Anti-REV-ERBα DOX 1 μg/mL (cont.)

Chemiluminescence

Biological replicate 2

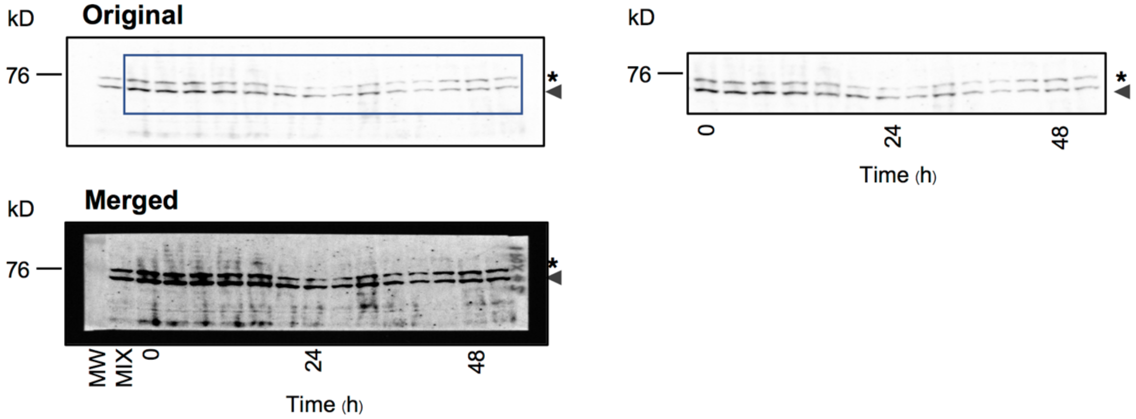

Biological replicate 3

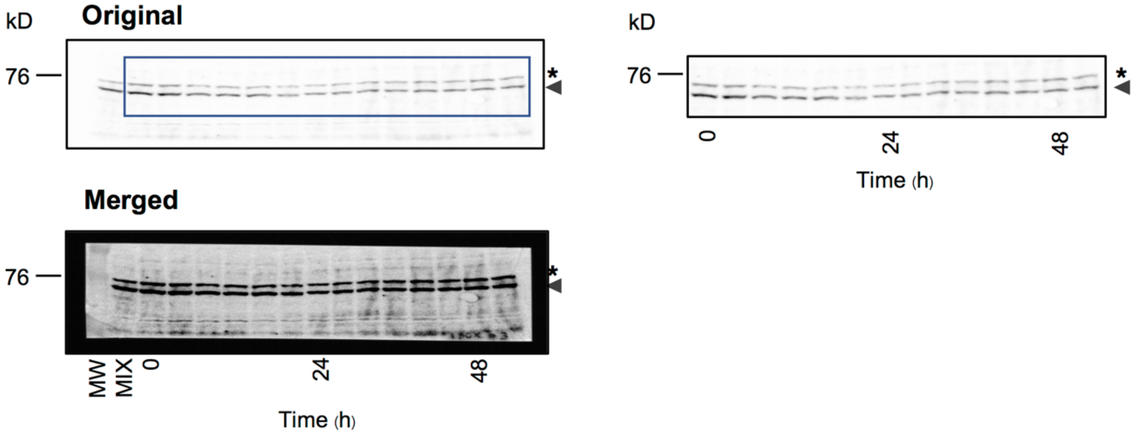

Fig 4D Anti-CLOCK DOX 0.1 μg/mL

Total protein

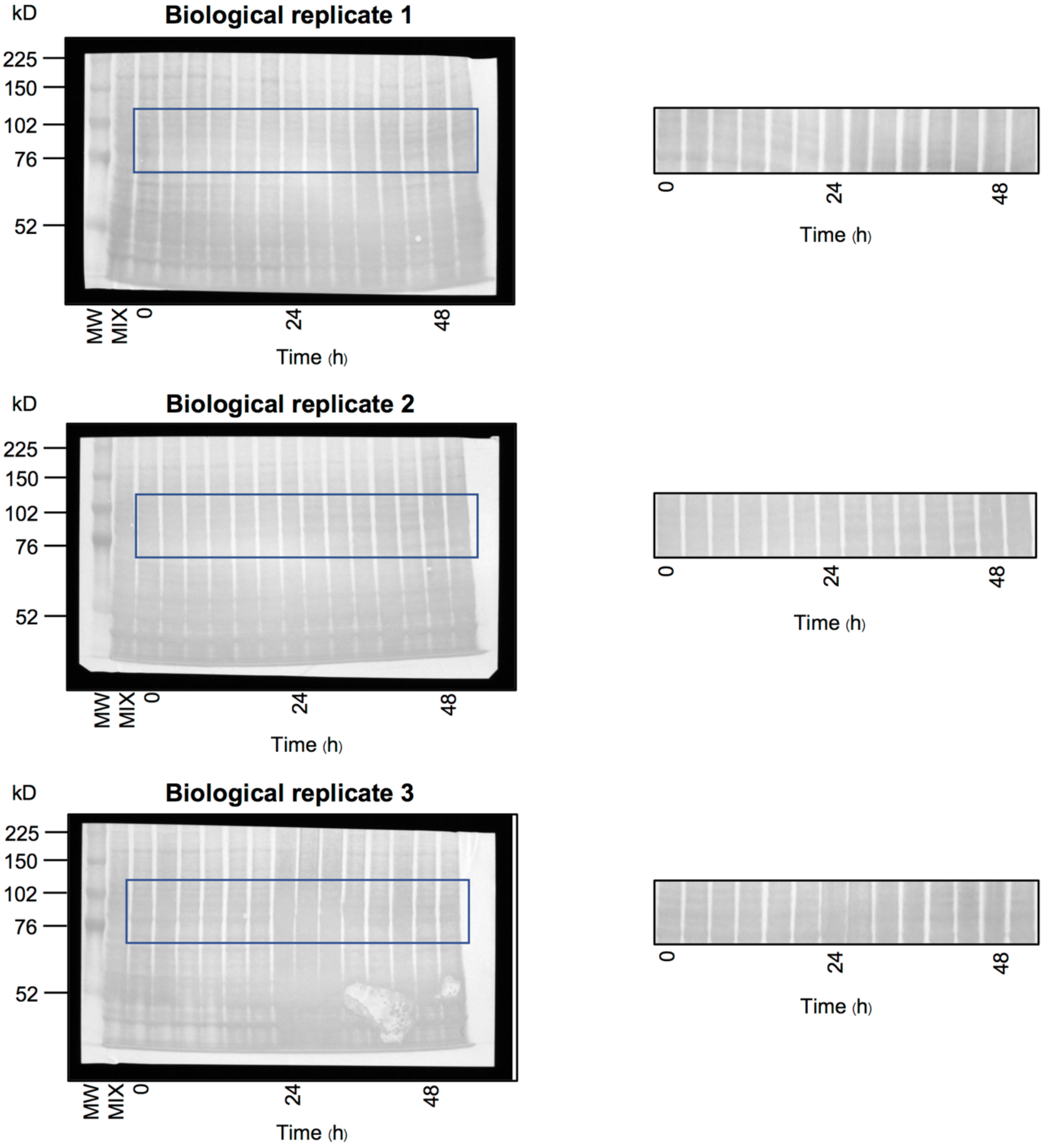

Figure continued on next page

Fig 4D Anti-CLOCK DOX 0.1 µg/mL (cont.)

Chemiluminescence

Biological replicate 1

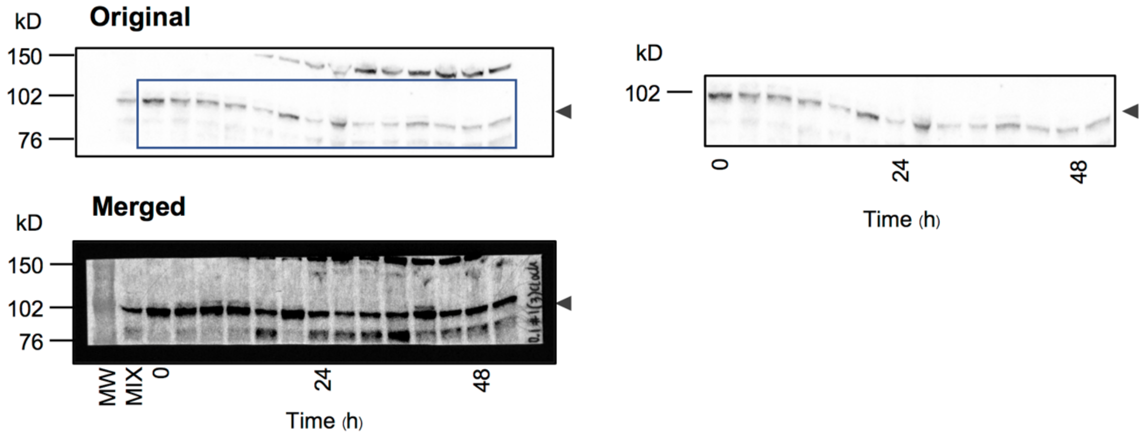

Biological replicate 2

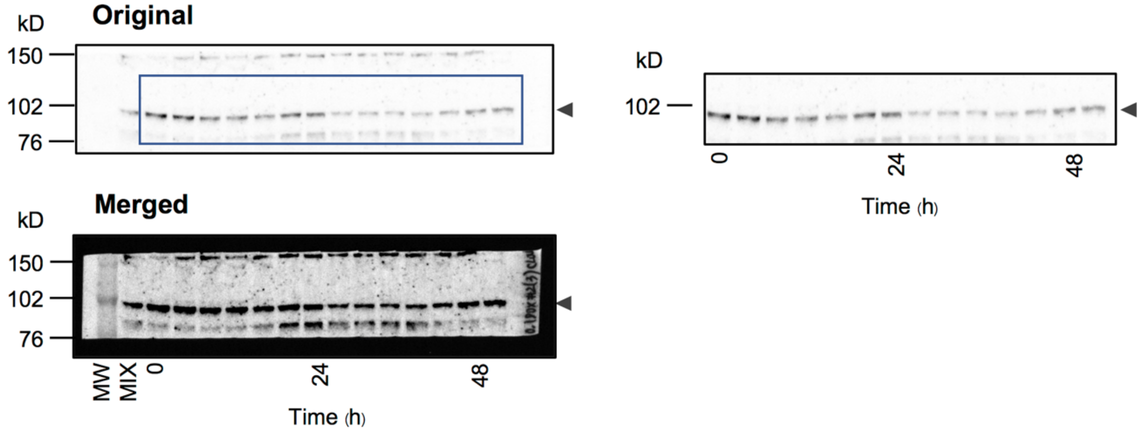

Biological replicate 3

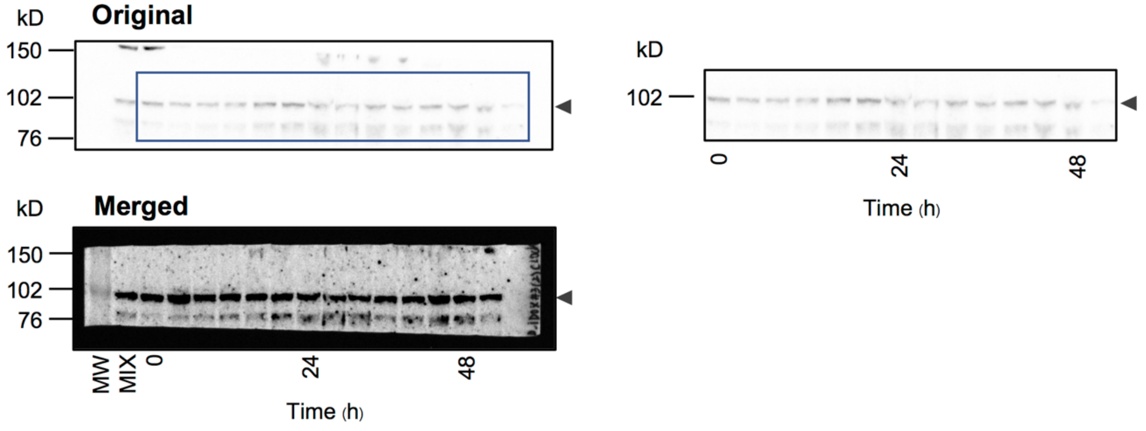

Fig 4E Anti-CLOCK DOX 1 μg/mL

Total protein

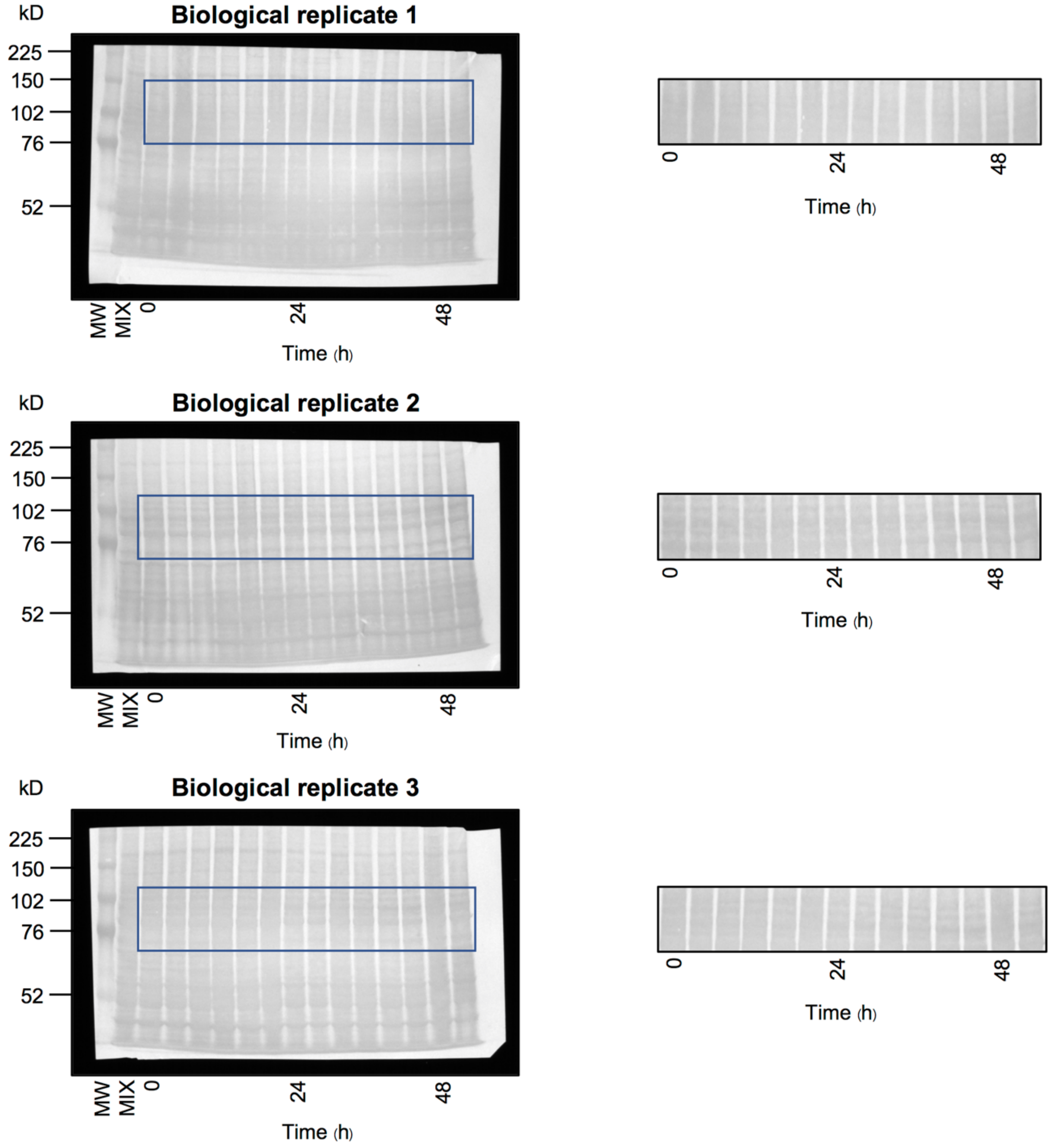

Figure continued on next page

Fig 4E Anti-CLOCK DOX 1 µg/mL (cont.)

Chemiluminescence

Biological replicate 1

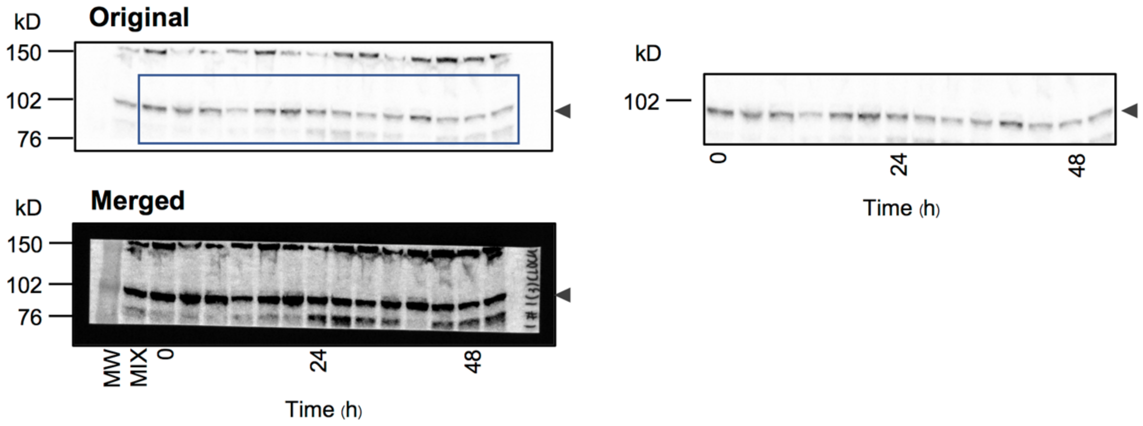

Biological replicate 2

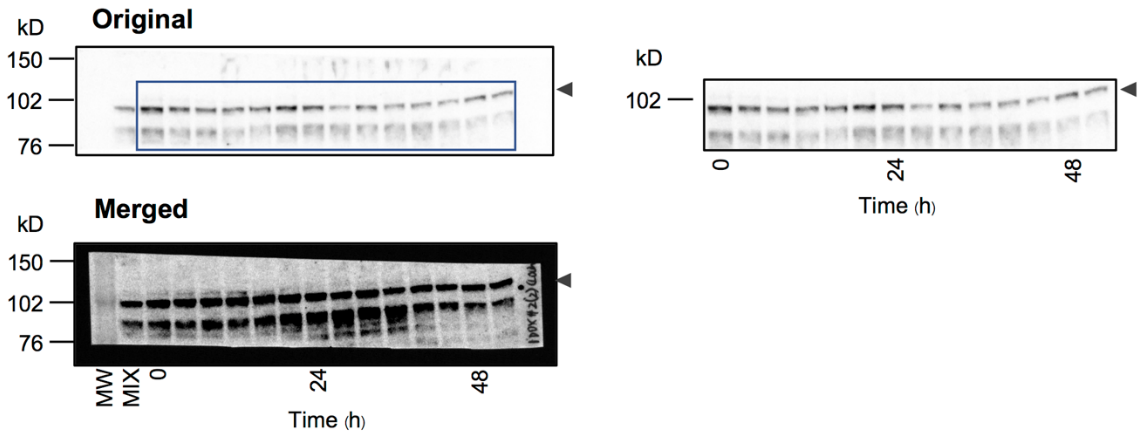

Biological replicate 3

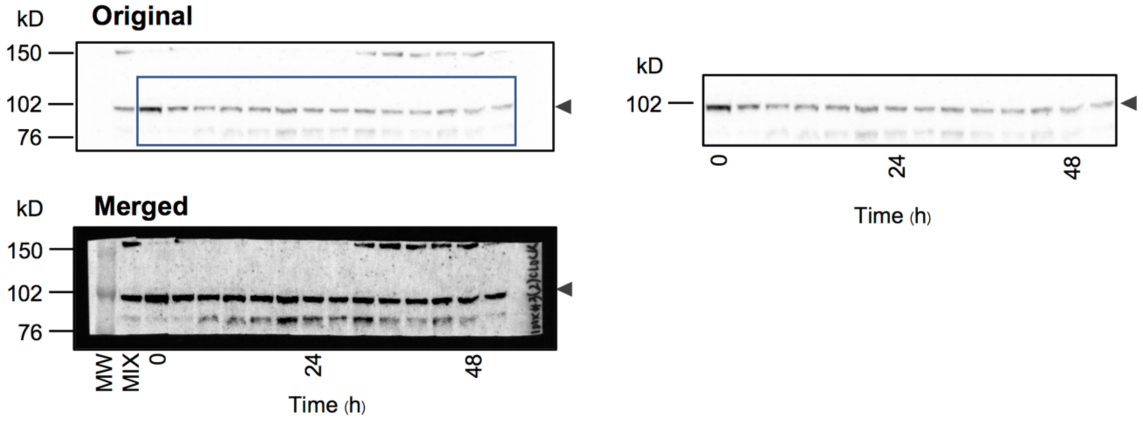

Fig. S2 Anti-Myc (Strain-2)

Total protein

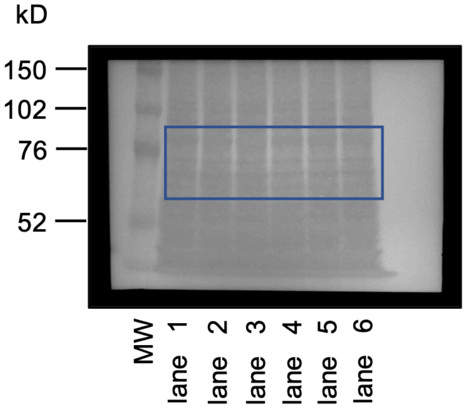

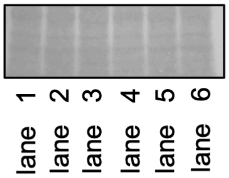

Chemiluminescence

Original

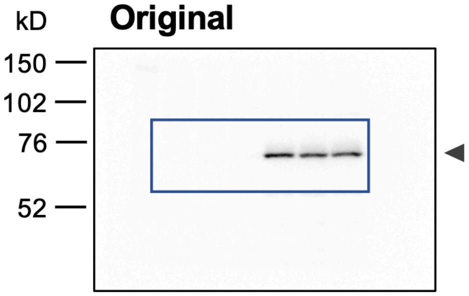

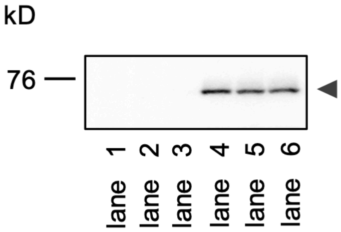

Merged

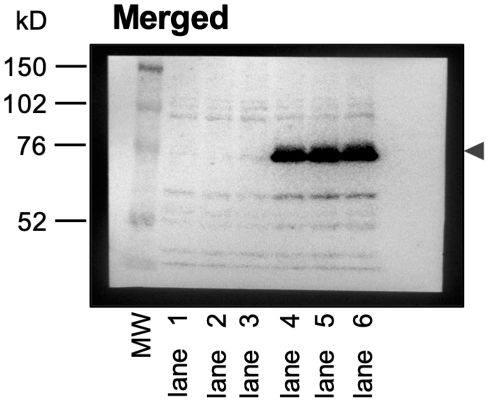

lane 1 : Myc-Bmal1 strain-2 DOX 0.01 μg/mL Biological replicate 1
lane 2 : Myc-Bmal1 strain-2 DOX 0.01 μg/mL Biological replicate 2
lane 3 : Myc-Bmal1 strain-2 DOX 0.01 μg/mL Biological replicate 3
lane 4 : Myc-Bmal1 strain-2 DOX 1 μg/mL Biological replicate 1
lane 5 : Myc-Bmal1 strain-2 DOX 1 μg/mL Biological replicate 2
lane 6 : Myc-Bmal1 strain-2 DOX 1 μg/mL Biological replicate 3

Fig. S2 Anti-Myc (Strain-17 and -23)

Total protein

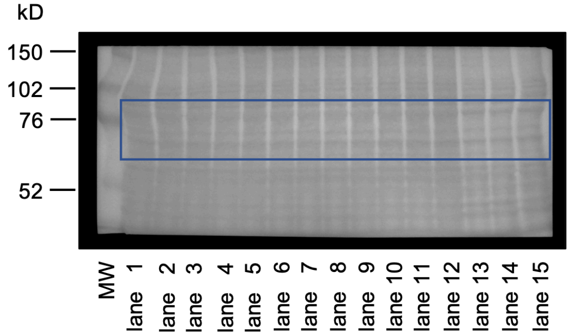

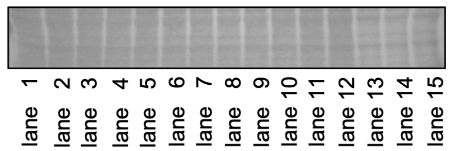

Chemiluminescence

Original

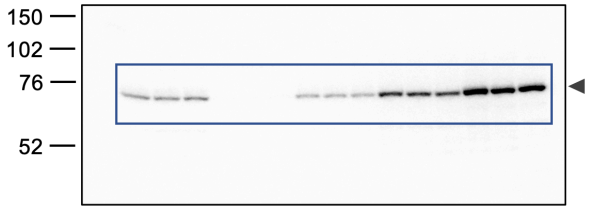

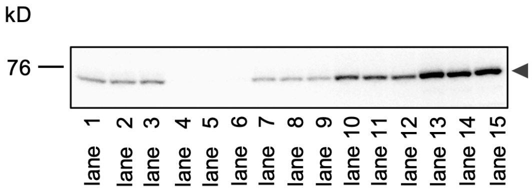

Merged

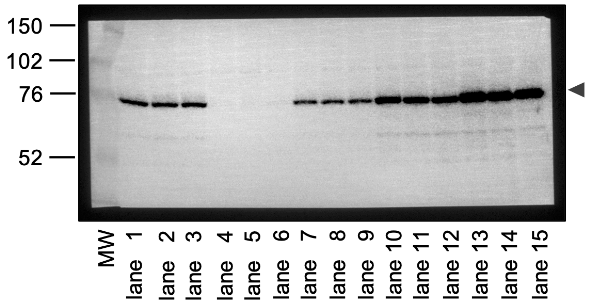

lane 1 : Myc-Bmal1 strain-2 DOX 1 μg/mL Biological replicate 1
lane 2 : Myc-Bmal1 strain-2 DOX 1 μg/mL Biological replicate 2
lane 3 : Myc-Bmal1 strain-2 DOX 1 μg/mL Biological replicate 3
lane 4 : Myc-Bmal1 strain-17 DOX 0.01 μg/mL Biological replicate 1
lane 5 : Myc-Bmal1 strain-17 DOX 0.01 μg/mL Biological replicate 2
lane 6 : Myc-Bmal1 strain-17 DOX 0.01 μg/mL Biological replicate 3
lane 7 : Myc-Bmal1 strain-17 DOX 1 μg/mL Biological replicate 1
lane 8 : Myc-Bmal1 strain-17 DOX 1 μg/mL Biological replicate 2
lane 9 : Myc-Bmal1 strain-17 DOX 1 μg/mL Biological replicate 3
lane 10 : Myc-Bmal1 strain-23 DOX 0.01 μg/mL Biological replicate 1
lane 11 : Myc-Bmal1 strain-23 DOX 0.01 μg/mL Biological replicate 2
lane 12 : Myc-Bmal1 strain-23 DOX 0.01 μg/mL Biological replicate 3
lane 13 : Myc-Bmal1 strain-23 DOX 1 μg/mL Biological replicate 1
lane 14 : Myc-Bmal1 strain-23 DOX 1 μg/mL Biological replicate 2
lane 15 : Myc-Bmal1 strain-23 DOX 1 μg/mL Biological replicate 3

Fig. S2 Anti-Myc (Strain-27 and -33)

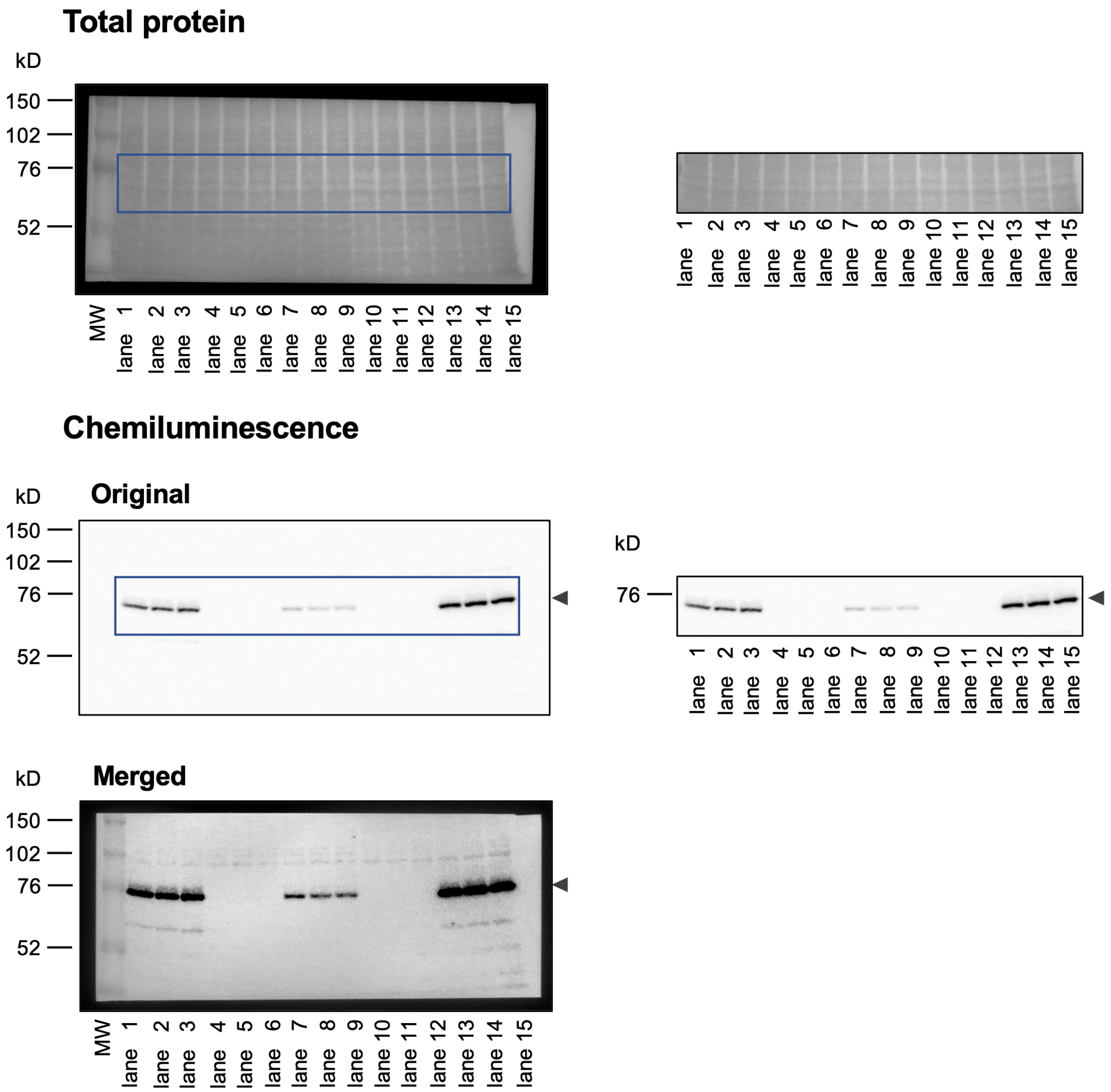

lane 1 : Myc-Bmal1 strain-2 DOX 1 µg/mL Biological replicate 1
lane 2 : Myc-Bmal1 strain-2 DOX 1 µg/mL Biological replicate 2
lane 3 : Myc-Bmal1 strain-2 DOX 1 µg/mL Biological replicate 3
lane 4 : Myc-Bmal1 strain-27 DOX 0.01 µg/mL Biological replicate 1
lane 5 : Myc-Bmal1 strain-27 DOX 0.01 µg/mL Biological replicate 2
lane 6 : Myc-Bmal1 strain-27 DOX 0.01 µg/mL Biological replicate 3
lane 7 : Myc-Bmal1 strain-27 DOX 1 µg/mL Biological replicate 1
lane 8 : Myc-Bmal1 strain-27 DOX 1 µg/mL Biological replicate 2
lane 9 : Myc-Bmal1 strain-27 DOX 1 µg/mL Biological replicate 3
lane 10 : Myc-Bmal1 strain-33 DOX 0.01 µg/mL Biological replicate 1
lane 11 : Myc-Bmal1 strain-33 DOX 0.01 µg/mL Biological replicate 2
lane 12 : Myc-Bmal1 strain-33 DOX 0.01 µg/mL Biological replicate 3
lane 13 : Myc-Bmal1 strain-33 DOX 1 µg/mL Biological replicate 1
lane 14 : Myc-Bmal1 strain-33 DOX 1 µg/mL Biological replicate 2
lane 15 : Myc-Bmal1 strain-33 DOX 1 µg/mL Biological replicate 3

Fig. S2 Anti-Myc (Strain-51 and -59)

Total protein

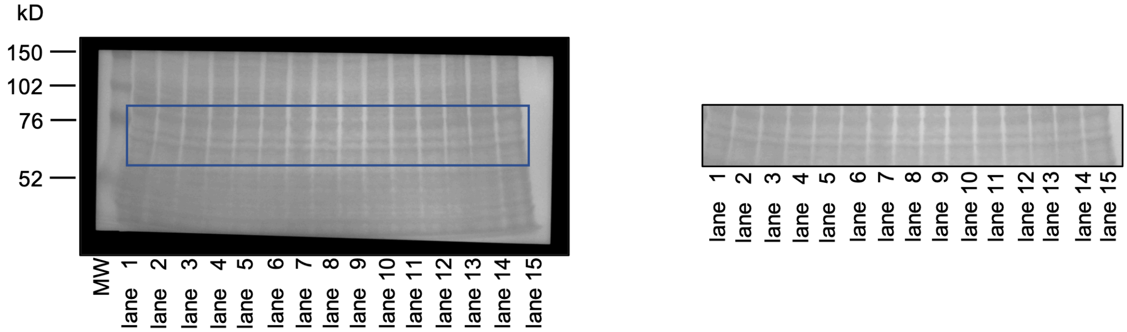

Chemiluminescence

Original

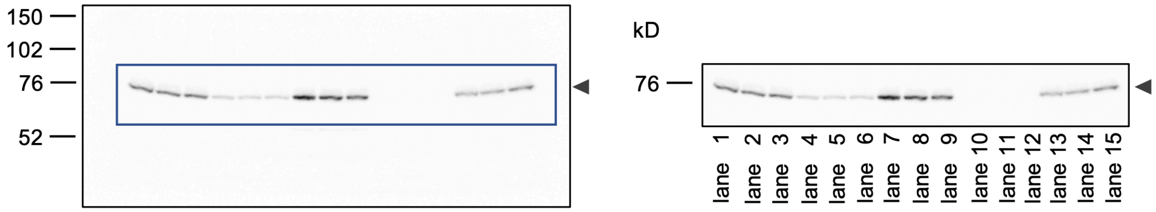

Merged

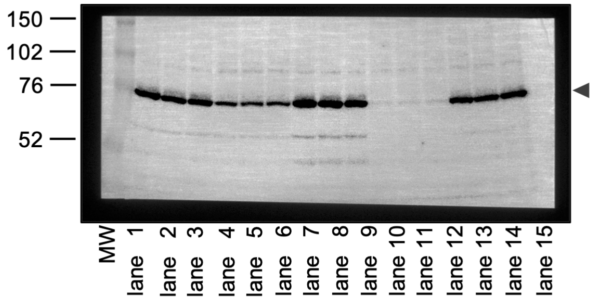

lane 1 : Myc-Bmal1 strain-2 DOX 1 μg/mL Biological replicate 1
lane 2 : Myc-Bmal1 strain-2 DOX 1 μg/mL Biological replicate 2
lane 3 : Myc-Bmal1 strain-2 DOX 1 μg/mL Biological replicate 3
lane 4 : Myc-Bmal1 strain-51 DOX 0.01 μg/mL Biological replicate 1
lane 5 : Myc-Bmal1 strain-51 DOX 0.01 μg/mL Biological replicate 2
lane 6 : Myc-Bmal1 strain-51 DOX 0.01 μg/mL Biological replicate 3
lane 7 : Myc-Bmal1 strain-51 DOX 1 μg/mL Biological replicate 1
lane 8 : Myc-Bmal1 strain-51 DOX 1 μg/mL Biological replicate 2
lane 9 : Myc-Bmal1 strain-51 DOX 1 μg/mL Biological replicate 3
lane 10 : Myc-Bmal1 strain-59 DOX 0.01 μg/mL Biological replicate 1
lane 11 : Myc-Bmal1 strain-59 DOX 0.01 μg/mL Biological replicate 2
lane 12 : Myc-Bmal1 strain-59 DOX 0.01 μg/mL Biological replicate 3
lane 13 : Myc-Bmal1 strain-59 DOX 1 μg/mL Biological replicate 1
lane 14 : Myc-Bmal1 strain-59 DOX 1 μg/mL Biological replicate 2
lane 15 : Myc-Bmal1 strain-59 DOX 1 μg/mL Biological replicate 3

Fig. S4 Anti-BMAL1

Total protein

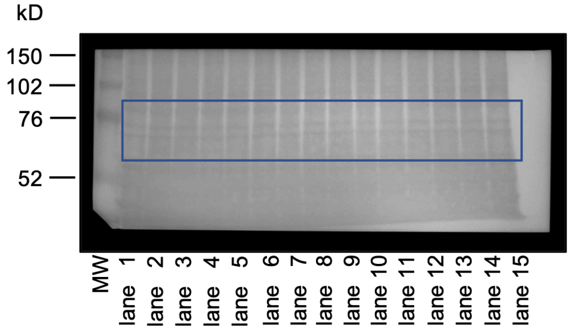

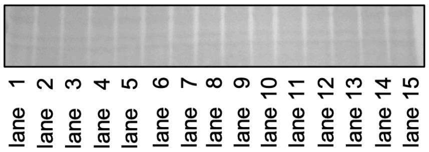

Chemiluminescence

Original

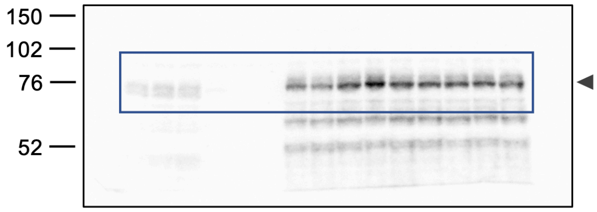

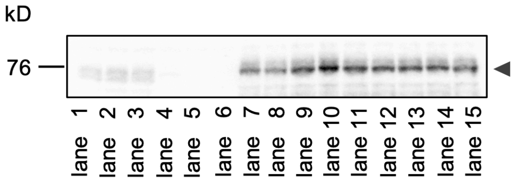

Merged

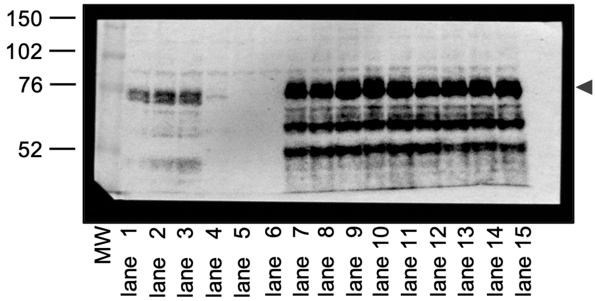

lane 1 : Bmal1 WT at 0-52 hours mixture Biological replicate 1
lane 2 : Bmal1 WT at 0-52 hours mixture Biological replicate 2
lane 3 : Bmal1 WT at 0-52 hours mixture Biological replicate 3
lane 4 : Myc-Bmal1 strain-2 DOX 0.01 μg/mL Biological replicate 1
lane 5 : Myc-Bmal1 strain-2 DOX 0.01 μg/mL Biological replicate 2
lane 6 : Myc-Bmal1 strain-2 DOX 0.01 μg/mL Biological replicate 3
lane 7 : Myc-Bmal1 strain-2 DOX 0.1 μg/mL Biological replicate 1
lane 8 : Myc-Bmal1 strain-2 DOX 0.1 μg/mL Biological replicate 2
lane 9 : Myc-Bmal1 strain-2 DOX 0.1 μg/mL Biological replicate 3
lane 10 : Myc-Bmal1 strain-2 DOX 1 μg/mL Biological replicate 1
lane 11 : Myc-Bmal1 strain-2 DOX 1 μg/mL Biological replicate 2
lane 12 : Myc-Bmal1 strain-2 DOX 1 μg/mL Biological replicate 3
lane 13 : Myc-Bmal1 strain-2 DOX 10 μg/mL Biological replicate 1
lane 14 : Myc-Bmal1 strain-2 DOX 10 μg/mL Biological replicate 2
lane 15 : Myc-Bmal1 strain-2 DOX 10 μg/mL Biological replicate 3

Fig. S6 Anti-REV-ERBα (WT U2OS)

Total protein

Biological replicate 1

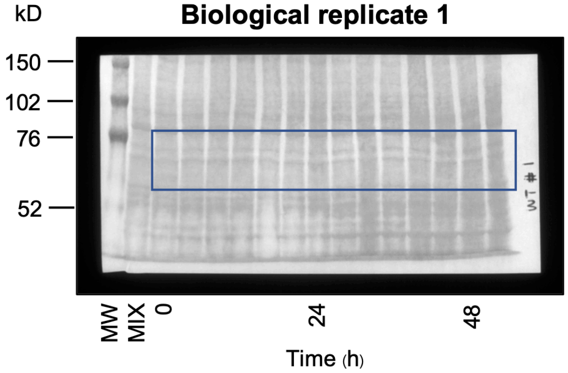

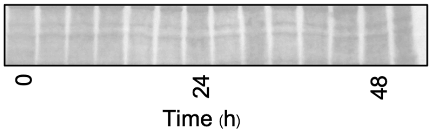

Biological replicate 2

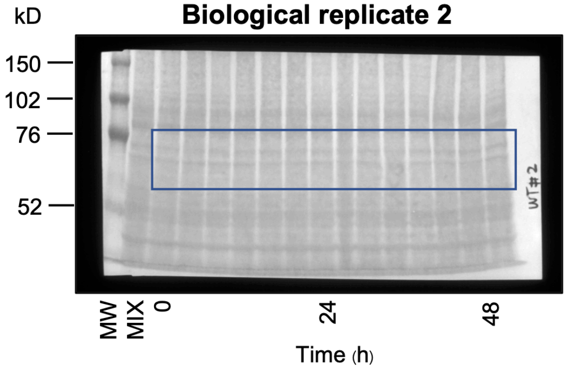

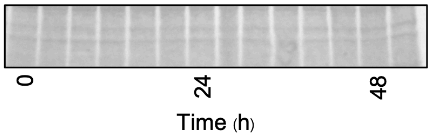

Biological replicate 3

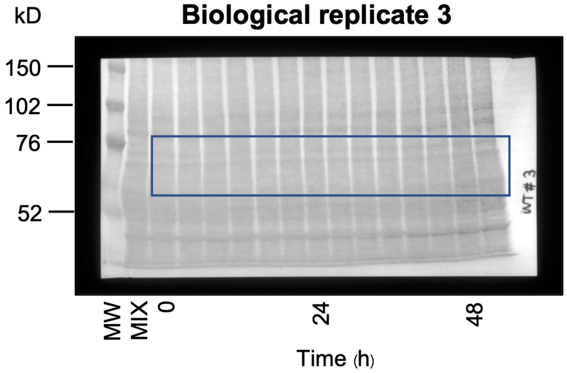

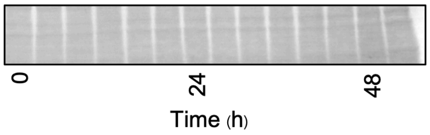

Figure continued on next page

Fig. S6 Anti-REV-ERBα (WT U2OS) (cont.)

Chemiluminescence

Biological replicate 1

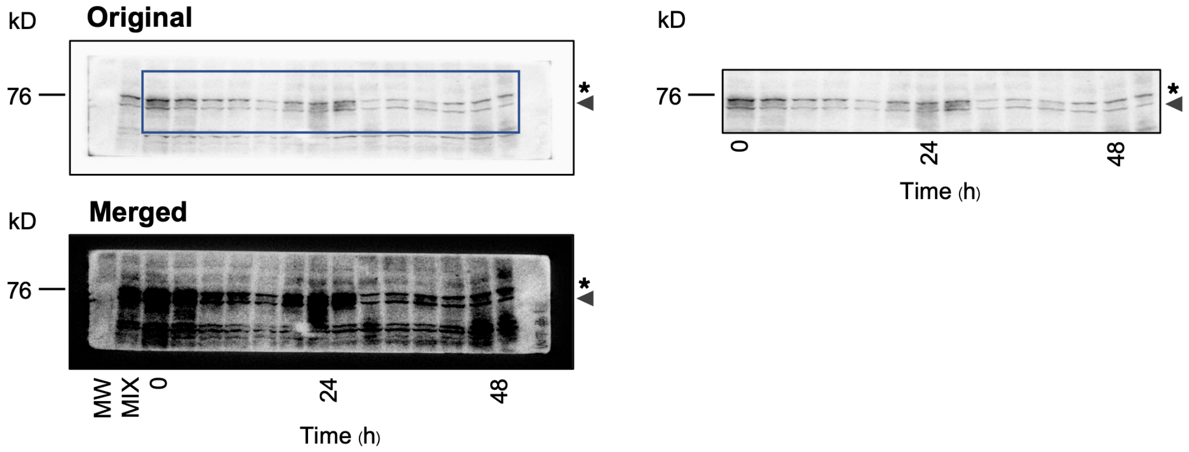

Biological replicate 2

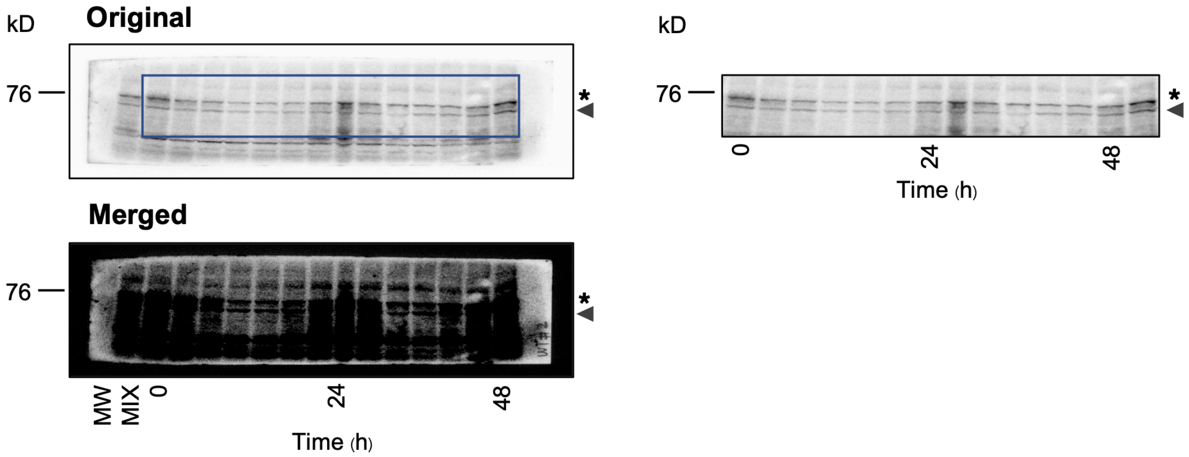

Biological replicate 3

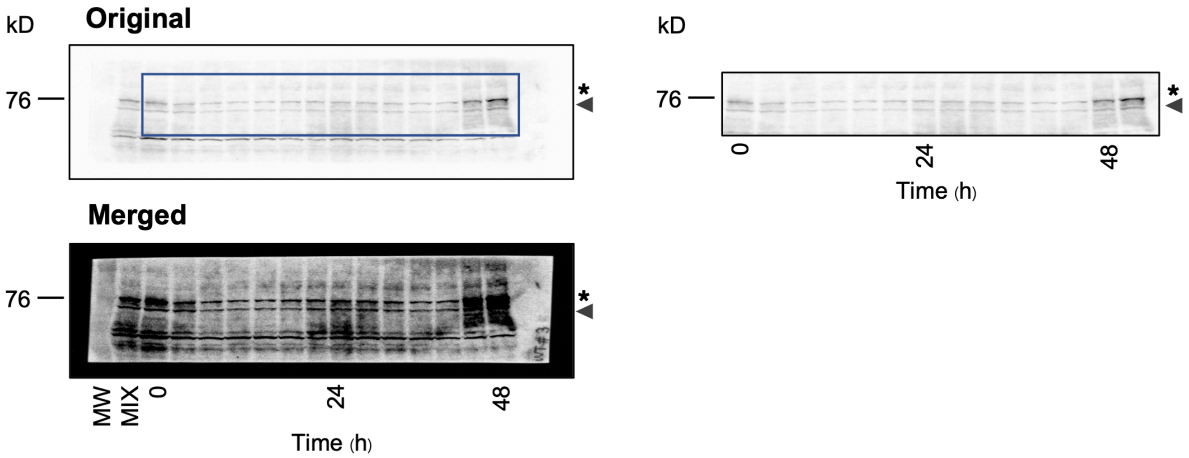

Supplement: Supplementary file 1 — Supplementary Information. [file 41598_2022_24188_MOESM1_ESM.pdf]
